# Reconstitution of purified membrane protein dimers in lipid nanodiscs with defined stoichiometry and orientation using a split GFP tether

**DOI:** 10.1016/j.jbc.2022.101628

**Published:** 2022-01-22

**Authors:** Elise S. Bruguera, Jacob P. Mahoney, William I. Weis

**Affiliations:** Departments of Molecular & Cellular Physiology and Structural Biology, Stanford University School of Medicine, Stanford, California, USA

**Keywords:** dimerization, G protein–coupled receptor, GFP, high-density lipoprotein, membrane protein, membrane reconstitution, nanodisc, protein purification, receptor, wnt signaling, BacMam, baculovirus for mammalian cells, BSA, bovine serum albumin, CHS, cholesteryl hemisuccinate, CV, column volume, DDM, *n*-dodecyl-β-d-maltopyranoside, DEP, contraction of Dishevelled, Egl-10, and Pleckstrin, DKK1, Dikkopf1, FKBP, the 12-kDa FK506-binding protein, Dvl2, Dishevelled-2, FSEC, fluorescence size-exclusion chromatography, Fzd, Frizzled, GFPnb, GFP nanobody, GPCR, G-protein–coupled receptor, HNE, Hepes, NaCl, and EDTA, LRP5/6, low-density lipoprotein receptor–related protein 5 or 6, MBP, maltose-binding protein, MESD, mesoderm development candidate 2, MOI, multiplicity of infection, PDZ, contraction of postsynaptic density protein 95, *Drosophila* disc large tumor suppressor, and zonula occludens-1, PIP2, phosphatidylinositol-4,5-bisphosphate, POPC, phosphatidylcholine, SEC, size-exclusion chromatography, sGFP, split GFP, sGFP1–10, an engineered version of the first 10 strands of the GFP beta barrel, sGFP11, an engineered 11th and final strand of the GFP beta barrel

## Abstract

Many membrane proteins function as dimers or larger oligomers, including transporters, channels, certain signaling receptors, and adhesion molecules. In some cases, the interactions between individual proteins may be weak and/or dependent on specific lipids, such that detergent solubilization used for biochemical and structural studies disrupts functional oligomerization. Solubilized membrane protein oligomers can be captured in lipid nanodiscs, but this is an inefficient process that can produce stoichiometrically and topologically heterogeneous preparations. Here, we describe a technique to obtain purified homogeneous membrane protein dimers in nanodiscs using a split GFP (sGFP) tether. Complementary sGFP tags associate to tether the coexpressed dimers and control both stoichiometry and orientation within the nanodiscs, as assessed by quantitative Western blotting and negative-stain EM. The sGFP tether confers several advantages over other methods: it is highly stable in solution and in SDS-PAGE, which facilitates screening of dimer expression and purification by fluorescence, and also provides a dimer-specific purification handle for use with GFP nanobody–conjugated resin. We used this method to purify a Frizzled-4 homodimer and a Frizzled-4/low-density lipoprotein receptor–related protein 6 heterodimer in nanodiscs. These examples demonstrate the utility and flexibility of this method, which enables subsequent mechanistic molecular and structural studies of membrane protein pairs.

Integral membrane proteins play critical roles in cell signaling, molecular transport, and adhesion. Detailed mechanistic insight into membrane protein function at a molecular level requires biochemically pure proteins, which are typically extracted in detergent for biophysical or structural studies. Detergents, while efficient and relatively cost effective, have drawbacks, as they generally have shorter alkyl chains and their headgroups are chemically distinct from those of native membrane lipids, giving them distinct physicochemical properties that can affect function of the embedded membrane protein (*e.g.*, (1, 2)). In addition, many membrane proteins are known to form homodimers or heterodimers or larger oligomers (3), which are sometimes mediated by specific lipids (4, 5, 6, 7). In some cases, such as class A G-protein–coupled receptors (GPCRs), oligomerization is transient or weak (8), and intramembrane interactions are disrupted during detergent extraction. In these cases, experiments using purified proteins in detergent micelles are often limited to the monomeric state. Reconstitution into liposomes can provide a quasi-native environment for mechanistic studies of membrane proteins, including the role of oligomerization. However, liposomes vary in size and number of receptors, the receptor orientation is not controlled or uniform, and individual receptors are only accessible on the extravesicular side. These factors limit the use of liposomes for structural and some functional analyses.

These considerations show that a method to obtain pure membrane protein homooligomers and heterooligomers in a lipid environment is crucial for understanding molecular mechanisms governing receptors, transporters, adhesion proteins, and other membrane proteins that function in complexes. To this end, membrane proteins have also been reconstituted into high-density lipoprotein particles (nanodiscs) (9), which offer several advantages compared with liposome reconstitution. Specifically, nanodisc-embedded membrane proteins are accessible from both sides of the lipid bilayer, are homogeneous, and are amenable to structure determination by single-particle cryo-EM. Nanodiscs have often been used to study monomeric membrane proteins, but reconstitution of oligomeric proteins in nanodiscs can be challenging. Dimers have been reconstituted by increasing the ratio of membrane protein to scaffold protein during reconstitution (10), and heterodimers can be isolated by sequential purification with affinity tags (*e.g.*, (11)). However, these methods rely on high dimer affinity and often result in heavy protein losses. Critically, the composition, stoichiometry, and relative orientation of the proteins within each nanodisc cannot be controlled, making experiments with these heterogeneous protein complexes difficult to interpret.

Here, we describe an approach to purify defined, stoichiometric, and parallel homodimeric or heterodimeric membrane protein complexes in nanodiscs. In this method, two membrane proteins are tethered together by complementary fragments of split GFP (sGFP) (12): sGFP1–10 (an engineered version of the first 10 strands of the GFP beta-barrel) and sGFP11 (an engineered 11th and final strand). sGFP-tagged receptors are coexpressed, copurified, and inserted into nanodiscs for biophysical and structural studies. We show two examples using cell surface receptors that mediate Wnt/β-catenin signaling. In this pathway, secreted Wnt ligands bind to Frizzled (Fzd), a member of the GPCR superfamily, and the single-pass receptor low-density lipoprotein receptor–related protein 5 or 6 (LRP5/6). Ligand binding leads to the stabilization of the transcriptional coactivator β-catenin and expression of Wnt target genes. Prior studies have suggested that ligand-induced homodimerization and heterodimerization of these receptors initiates signaling, so we prepared Fzd4 homodimers as well as Fzd4–LRP6 heterodimers in order to understand the contribution of receptor dimerization to the initial steps of Wnt/β-catenin signal transduction.

## Results

**Figure 1:**
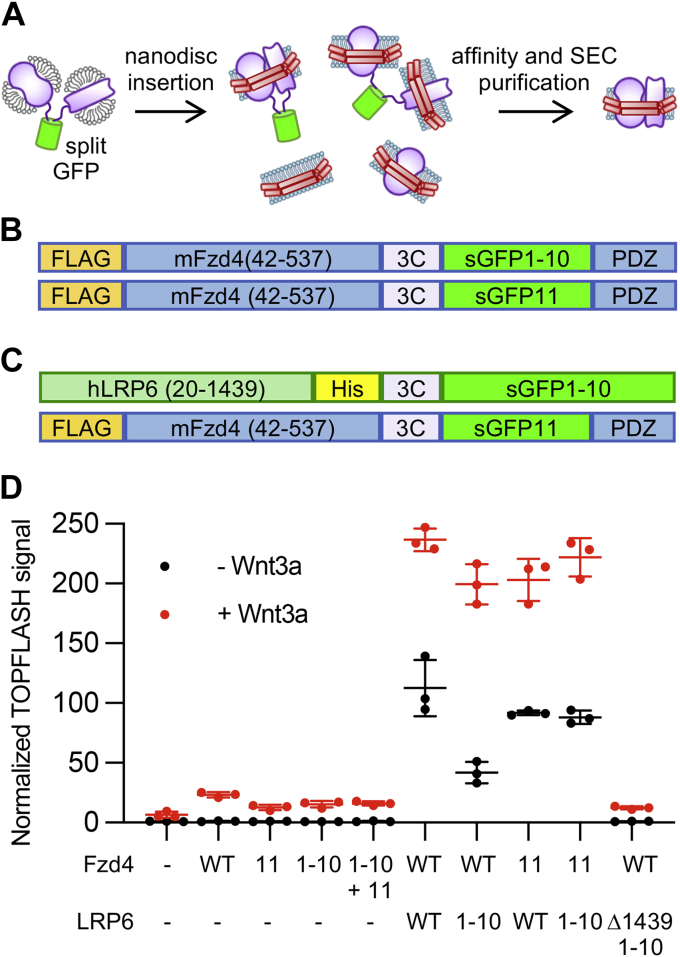
**Method overview, construct design, and functional testing.***A*, in this method, receptors are tagged with complementary fragments of split GFP (sGFP) and coexpressed to form defined GFP-tethered dimers. Dimers are inserted into nanodiscs, after which the GFP moiety is proteolytically cleaved, and the desired nanodisc-embedded dimers are isolated by size-exclusion and affinity chromatography. *B*, to form Fzd4 homodimer, full-length Fzd4 constructs were expressed using a hemagglutinin signal sequence followed by an N-terminal FLAG epitope. Mouse Fzd4 (residues 42–537) was followed by a 3C protease cleavage site and one of the two (s1–10 or s11) sGFP fragments. The last four residues of *wt* Fzd4, which are a PDZ ligand (“PDZ”) and were found to enhance expression (Fig. 2, *A* and *B*), were fused to the C terminus. *C*, Fzd4–LRP6 heterodimer formation employed a Fzd4 construct identical to that in the homodimer in (*B*). The signal sequence of LRP6 was replaced with the hemagglutinin signal sequence, and LRP6 was truncated after residue 1439. The LRP6 construct contained a His_8_ tag followed by a 3C protease cleavage site and the sGFP fragment. *D*, constructs were tested for their ability to stabilize β-catenin in response to Wnt3a. Wildtype HEK293 cells were transfected with empty vector or indicated Fzd4 and LRP6 constructs along with a luciferase reporter downstream of a β-catenin–responsive element (SuperTOPFLASH) and a LacZ plasmid as a control, stimulated with control or Wnt3a-conditioned media. Luciferase signal normalized to LacZ and then to the unstimulated receptor-less condition, ±SD for three wells is plotted and is representative of three independent experiments. For LRP6 transfections, full-length LRP6 with or without sGFP1–10 showed constitutive and Wnt-dependent activity; where the purification construct (Δ1439-sGFP1–10) is cotransfected with WT Fzd4, TOPFLASH signal is less than that of Fzd4 alone, as expected given that LRP6 lacking the C terminus is dominant negative (14). Fzd4, Frizzled-4; HEK293, human embryonic kidney 293 cell line; PDZ, contraction of postsynaptic density protein 95, *Drosophila* disc large tumor suppressor, and zonula occludens-1.

We developed a flexible method utilizing the self-assembly of sGFP fragments to generate defined membrane protein dimers (Fig. 1*A*). The sGFP moiety tethers the intracellular sides of the receptors, enforcing a parallel orientation when the complex is inserted into nanodiscs. It is also useful as a purification handle: intact complexes bearing folded GFP can be selectively purified using GFP nanobody (GFPnb)-conjugated resin (13). Assembled sGFP is highly stable, so the dimeric receptor complex stays intact throughout the purification process and in SDS-PAGE gels. The intact receptor–GFP complex can be detected by flow cytometry, fluorescence size-exclusion chromatography (FSEC), and SDS-PAGE in-gel fluorescence for facile construct screening.

### Construct design and expression optimization

**Figure 2:**
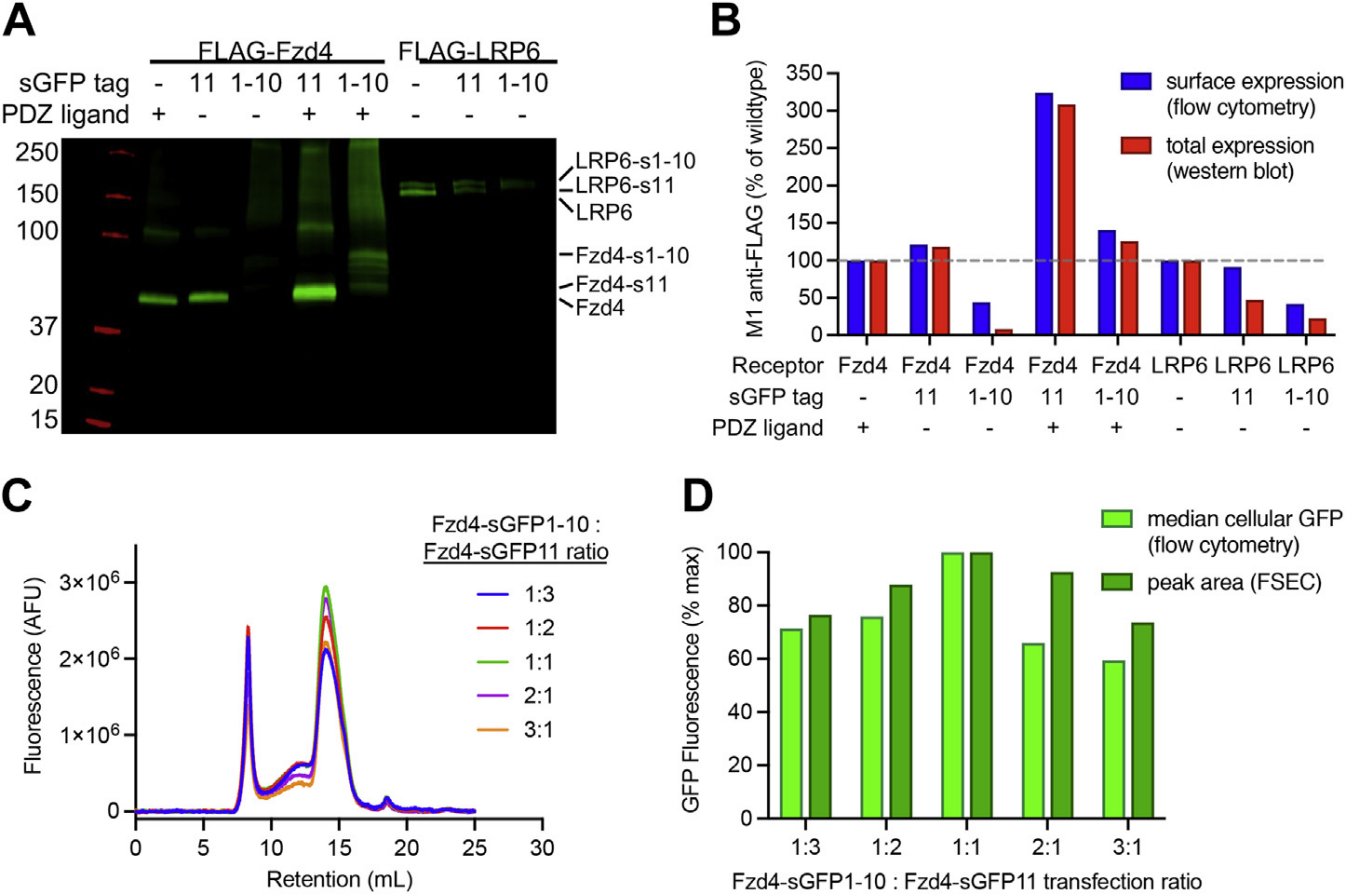
**Representative expression tests.***A*, M1 anti-FLAG Western blot of DDM-solubilized Expi293 cell membranes expressing the indicated Fzd4 and LRP6 constructs tagged with split GFP (sGFP) fragments. Restoring the last four residues of wildtype Fzd4 (ETVV, a PDZ ligand sequence) to the C terminus of the sGFP-fused constructs was found to boost expression of the Fzd4-sGFP constructs. *B*, quantification of surface and total expression of FLAG-tagged Fzd4 and LRP6-sGFP constructs. Surface expression was assessed by flow cytometry of cells labeled with Alexa Fluor 647-conjugated M1 anti-FLAG antibody. Median anti-FLAG fluorescence is plotted alongside the quantified Western blot bands from (*A*). Data represent a single experiment. *C*, FSEC traces showing optimization of Fzd4-sGFP1–10 and Fzd4-sGFP11 cotransfection ratios. Constructs were transiently cotransfected at different ratios in 10 ml Expi293 cells per condition, with the total amount of transfected DNA held constant across conditions. Membranes were solubilized, clarified, and injected on a Superose 6 10/300 column. GFP fluorescence (487 nm excitation and 507 nm emission) is plotted for indicated ratios. *D*, normalized median cellular GFP fluorescence measured by flow cytometry for the transfected conditions from (*C*), plotted alongside normalized area of each 14 ml FSEC peak in (*C*). Data represent a single experiment. DDM, *n*-dodecyl-β-d-maltopyranoside; FSEC, fluorescence size-exclusion chromatography; Fzd4, Frizzled-4; LRP6, low-density lipoprotein receptor–related protein 6; PDZ, contraction of postsynaptic density protein 95, *Drosophila* disc large tumor suppressor, and zonula occludens-1.

Figure 1, *B* and *C* shows the design of the Fzd4 and LRP6 constructs used in this study. Both receptors possess flexible cytosolic C termini onto which the complementary sGFP tags were introduced. Total and cell surface expression of receptor–sGFP fusions was measured by Western blot and flow cytometry (Fig. 2, *A* and *B*). Optimal ratios at which to coexpress receptor constructs were assessed by GFP fluorescence intensity using flow cytometry and FSEC of solubilized membranes (Fig. 2, *C* and *D*). We found that the sGFP tags decreased expression of LRP6 about twofold, with a larger effect seen for the larger fragment (sGFP1–10). sGFP-tagged Fzd4 surface expression was initially impaired compared with wildtype Fzd4 but could be restored to at least wildtype levels when the four residues at the C terminus, which comprise a PDZ (contraction of postsynaptic density protein 95, *Drosophila* disc large tumor suppressor, and zonula occludens-1) ligand, were appended to the C terminus after the sGFP fragment.

Coexpression appeared to be necessary for complementation of the sGFP fragments. Proteins tagged with uncomplemented sGFP1–10 were poorly behaved when purified alone, and we found that complementation is inefficient when receptors are expressed and purified separately before mixing. This observation is consistent with the localization of sGFP1–10 to *Escherichia coli* inclusion bodies and a refolding requirement for complementation (12).

As expected, given that the receptor fusion constructs were expressed on the cell surface (Fig. 2*B*), cells expressing Fzd4 and full-length LRP6 receptor sGFP fusion constructs responded similarly to wildtype receptors to Wnt3a in a luciferase reporter assay monitoring β-catenin transcriptional activity (TOPFLASH (15); Fig. 1*D*).

### Fzd4 homodimer purification

**Figure 3:**
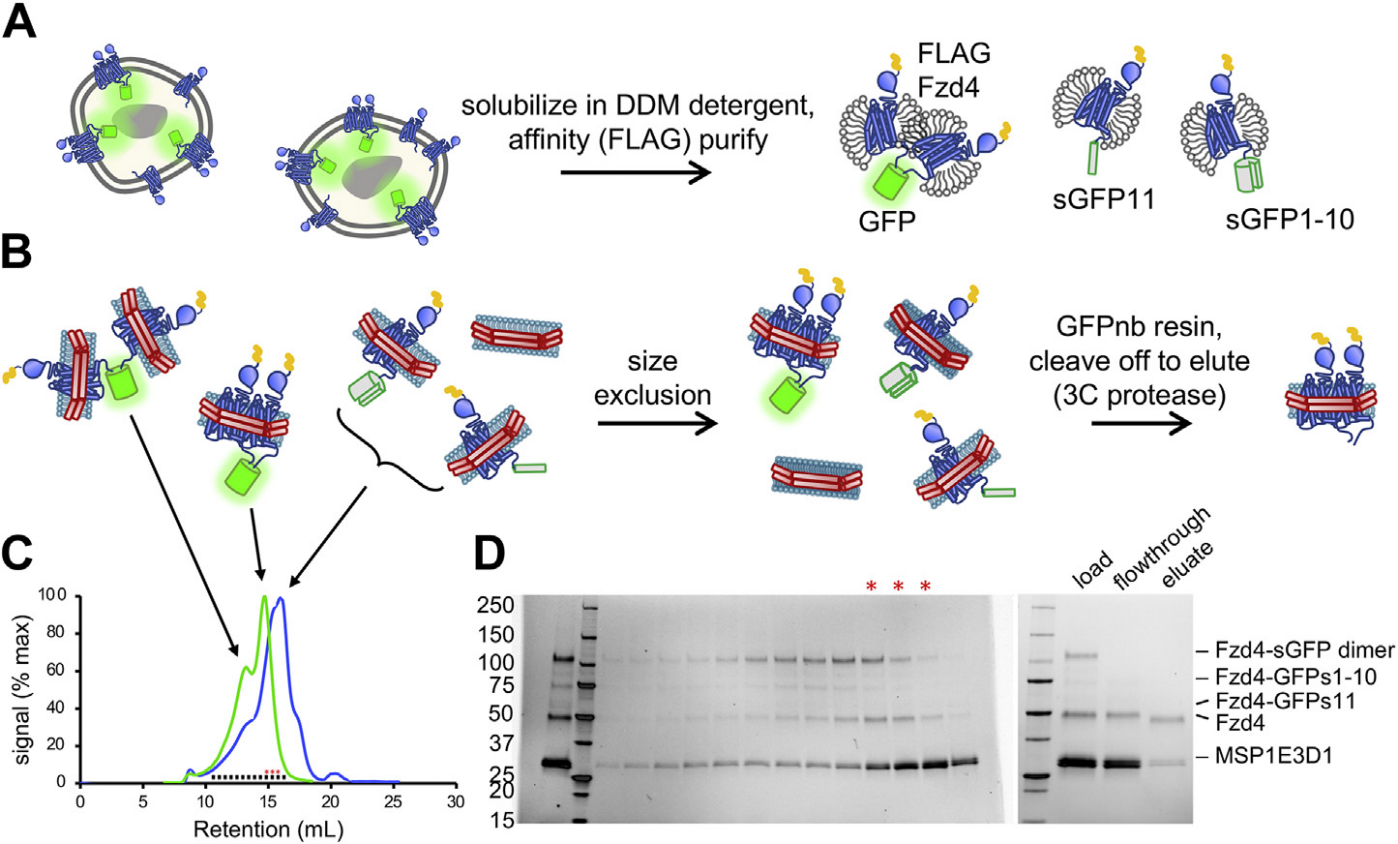
**Fzd4 homodimer reconstitution and purification.***A*, FLAG-Fzd4-sGFP1–10 and FLAG-Fzd4-sGFP11 were coexpressed in Sf9 cells, extracted in DDM, and purified on M1 anti-FLAG affinity resin to obtain both monomers and dimers. *B*, receptors were reconstituted into excess MSP1E3D1 nanodiscs, purified by SEC to separate GFP-dimerized species with each protomer in a separate nanodisc from nanodisc-embedded dimers (*arrows* in [*C*]), and finally affinity purified on GFPnb-conjugated resin, eluted by proteolytic cleavage of the GFP. *C*, SEC trace of reconstituted nanodiscs on a Superose 6 10/300 column. *Cartoons* of species in peaks are indicated. In addition to monitoring absorbance at 280 nm (*blue curve*), GFP fluorescence of fractions was measured using a plate reader and plotted (*green curve*). Fractions analyzed by SDS-PAGE and pooled for purification are indicated by *black squares* and *red asterisks*, respectively. *D*, *left*, equally spaced fractions indicated in (*C*) were analyzed by SDS-PAGE, along with the total injected reconstitution mixture (lane 1). *Red asterisks* indicate pooled fractions from the right side of the GFP fluorescence peak, which contains intact nanodisc-embedded Fzd4 dimer. *Right*, pooled fractions were purified with GFPnb-conjugated resin and eluted with 3C protease overnight, as shown by SDS-PAGE gel. DDM, *n*-dodecyl-β-d-maltopyranoside; Fzd4, Frizzled-4; GFPnb, GFP nanobody; SEC, size-exclusion chromatography.

FLAG-tagged Fzd4 constructs with cleavable complementary sGFP1–10 and sGFP11 tags were coexpressed, solubilized in *n*-dodecyl-β-d-maltopyranoside (DDM), and copurified on M1 anti-FLAG affinity resin (Fig. 3*A*). To obtain a dimeric complex in detergent, the eluate was purified by size-exclusion chromatography (SEC) to separate dimers from monomers (Fig. S1, *A* and *B*). By negative-stain EM, the GFP-dimerized Fzd4 receptors appeared to occupy separate micelles when in detergent (Fig. S1*D*). To obtain reconstituted dimers in nanodiscs, FLAG-purified Fzd4 receptors (containing monomeric and dimeric Fzd4) were reconstituted into excess nanodiscs. This resulted in a mixture of empty nanodiscs, nanodiscs containing Fzd4 monomers, nanodiscs with a Fzd4-GFP dimer (the desired product), and GFP-dimerized Fzd4 with each Fzd4 protomer in a separate nanodisc (Fig. 3*B*). On a Superose 6 column, this last species eluted before the nanodiscs containing Fzd4-GFP dimers (Fig. S2) but the desired Fzd4-GFP dimers did not separate as well from empty discs or Fzd4 monomer-containing discs (Fig. 3, *B*–*D*). Therefore, the nanodisc-embedded dimers were then isolated using GFPnb resin, which specifically recognizes intact GFP but not individual sGFP1–10 or sGFP11 tags. The empty discs and monomeric Fzd4 discs did not bind the resin, and the bound Fzd4 dimer was eluted by specific cleavage with 3C protease (Fig. 3*D*, *right*).

Comparing total protein and GFP fluorescence, we found that after detergent solubilization, approximately 50% of the expressed protein was GFP dimerized (*e.g.*, see representative peak heights of Fzd4 monomer *versus* dimer in Fig. S1*A*), although we expect this to be protein-dependent. We found that sGFP-tethered dimers remained intact following SDS-PAGE and could be detected by in-gel fluorescence, and assembled sGFP was stable for over an hour in SDS-PAGE sample buffer at room temperature (Fig. S1*E*). The in-gel stability of sGFP allowed for facile analysis of sample composition at any purification step. Thus, we used SDS-PAGE densitometry to determine that the final yield of purified reconstituted Fzd4 homodimer relative to initial affinity-purified sGFP dimer was 12.9% (Fig. S3, *A* and *B*).

### Fzd4–LRP6 heterodimer purification

**Figure 4:**
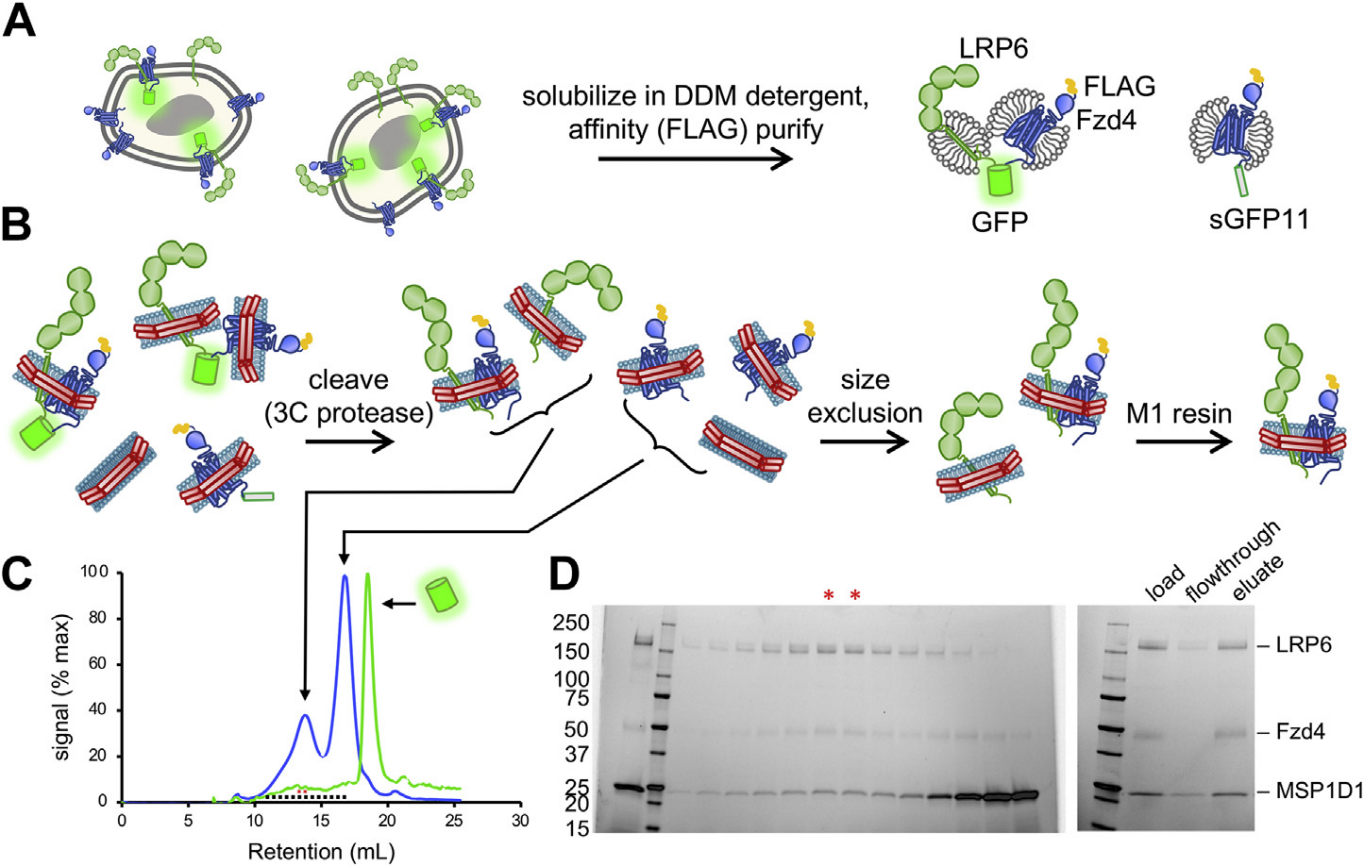
**Fzd4–LRP6 heterodimer purification.***A*, FLAG-Fzd4-sGFP11 and LRP-sGFP1–10 were coexpressed with MESD, an LRP5/6 chaperone, in Freestyle293 cells. The complex was solubilized in DDM and copurified on M1 anti-FLAG resin to obtain Fzd4 monomer as well as GFP-tethered dimer. *B*, nanodisc purification scheme. Receptor mixture was reconstituted into excess MSP1D1 nanodiscs and then cleaved with 3C protease before purification by size exclusion and M1 anti-FLAG affinity chromatography. *C*, a Superose 6 10/300 column separated discs containing LRP6 ± Fzd4 from empty discs or discs containing monomeric Fzd4, as indicated by *cartoons*. In addition to absorbance at 280 nm (*blue curve*), absorbance at 485 nm (*green curve*) was monitored to verify cleavage of GFP, which eluted later than the discs, as expected. Fractions analyzed by SDS-PAGE and pooled for purification are indicated by *black squares* and *red asterisks*, respectively. *D*, *left*, equally spaced fractions indicated in (*C*) were analyzed by SDS-PAGE, and peak fractions containing LRP6 (*red asterisks*) were pooled for M1 anti-FLAG purification of discs also containing Fzd4, as shown by SDS-PAGE gel at the *right*. DDM, *n*-dodecyl-β-d-maltopyranoside; Fzd4, Frizzled-4; LRP6, low-density lipoprotein receptor–related protein 6; MESD, full-length clone DNA of human mesoderm development candidate 2.

Fzd4–LRP6 heterodimer (Fig. 4) was purified similarly to Fzd4 homodimer, but some modifications were required, as the large size of the LRP6 extracellular domain prevented heterodimers embedded in a single nanodisc from separating from GFP-dimerized species with each protomer in a separate nanodisc by SEC. Therefore, the GFP tether was cleaved with 3C protease before SEC purification. The nanodisc-embedded heterodimer coeluted with monomeric LRP6 in nanodiscs but separated from Fzd4 monomer, empty nanodiscs, and free GFP. The peak heterodimer fractions were purified on M1 anti-FLAG affinity resin to isolate discs that contained FLAG-tagged Fzd4 to ensure recovery of heterodimer only. The final yield of purified and reconstituted Fzd4–LRP6 heterodimer relative to initial affinity-purified sGFP dimer was 8.3% (Fig. S3, *C* and *D*).

### Assessment of reconstituted dimer stoichiometry and topology

Receptor stoichiometry per nanodisc was measured using quantitative Western blotting, by comparing to standards of known concentration for each protein component. As expected, the stoichiometry in the samples was found to be approximately two Fzd4 receptors per nanodisc for Fzd4 homodimers and one Fzd4 and one LRP6 per disc for the Fzd4–LRP6 heterodimers (Table 1 and Fig. S4).

**Table 1:** Receptor stoichiometry by Western blot (±SD)

Complex	Receptor 1	Receptor 1:nanodisc ratio	Receptor 2	Receptor 2:nanodisc ratio
Fzd4 monomer^a^	Fzd4	1.34 ± 0.29	N/A	N/A
Fzd4 homodimer^b^	Fzd4	2.24 ± 0.25	N/A	N/A
Fzd4–LRP6 heterodimer^a^	Fzd4	0.98 ± 0.26	LRP6	0.81 ± 0.25

Abbreviation: N/A, not available.

a n = 3 independent reconstitutions, measured twice each.

b n = 5 independent reconstitutions, measured twice each.

**Figure 5:**
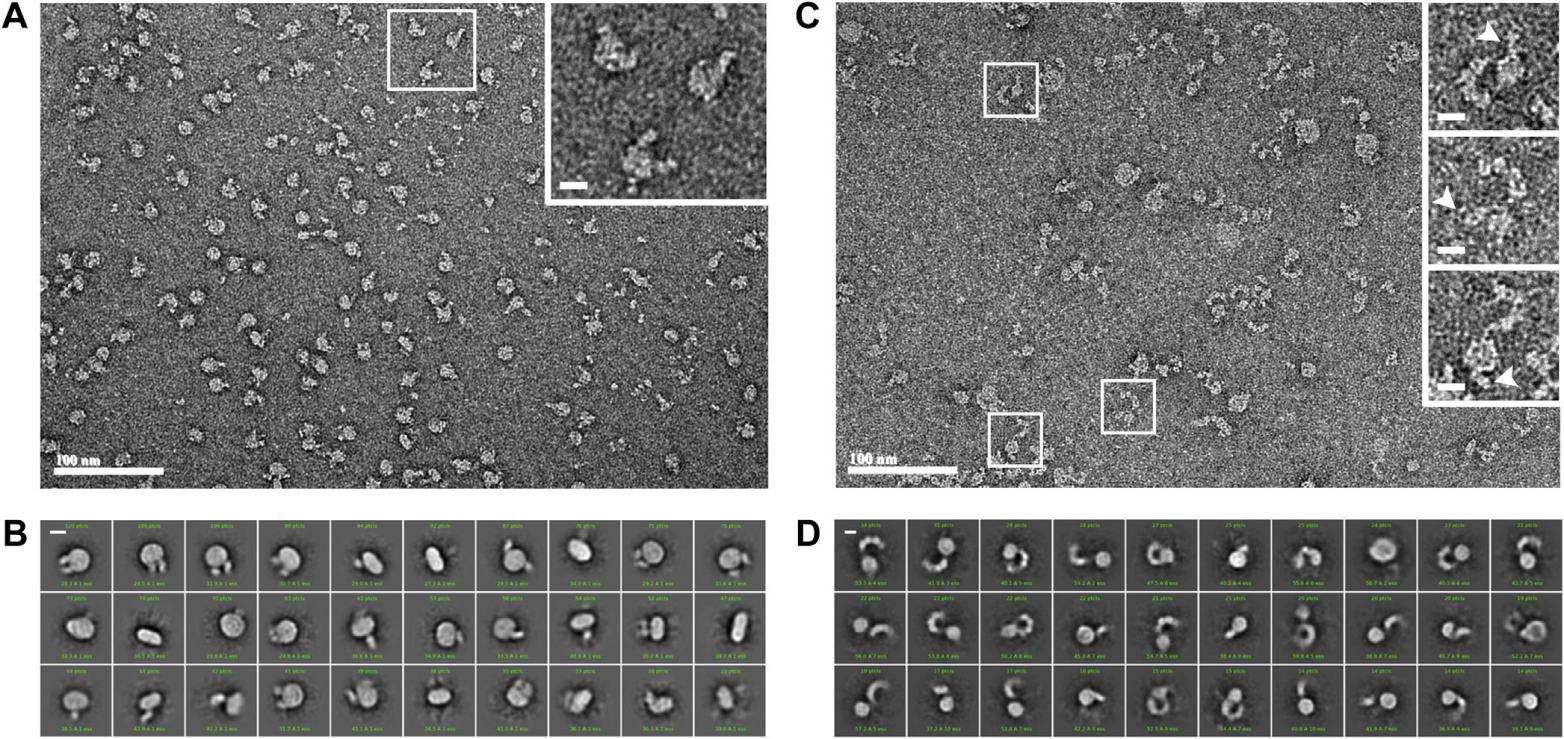
**Validation of parallel receptor orientation by negative-stain EM.***A*, representative negative-stain micrograph. *B*, 2D class averages of Fab-bound Fzd4 homodimer in nanodiscs. *C*, representative negative-stain micrograph and (*D*) 2D class averages of Fab-bound Fzd4–LRP6 heterodimer in nanodiscs. Representative particles are enlarged (*insets*); *white arrows* indicate visible Fabs. Both the homodimer and heterodimer discs appear to preferentially orient face up on the grid, and side views are rare. The scale bars in micrographs are 100 nm; the scale bars in *insets* and 2D class averages are 10 nm. Fzd4, Frizzled-4; LRP6, low-density lipoprotein receptor–related protein 6.

The relative orientation of Fzd4 receptor protomers within the reconstituted Fzd4 homodimers was examined using negative-stain EM with a Fab that binds the Fzd4 extracellular domain (16) as a fiducial marker (Fig. 5, *A* and *B*). Nanodiscs were preferentially oriented on the sample grid, with 2D averages dominated by face-on views and a minority of side views. The side-view 2D classes predominantly showed two Fabs on the same side of the nanodisc, indicating that the Fzd4 homodimers are reconstituted in a parallel orientation.

Fzd4–LRP6 heterodimers in complex with the Fzd4 Fab were also examined by negative-stain EM (Fig. 5, *C* and *D*). The LRP6 extracellular region dominated the 2D averages, and the Fab was not readily identifiable in the class averages. While the Fab was visible in some individual particles (Fig. 5*C*, *insets*), the position of the extracellular domains relative to the nanodisc could not be clearly determined because of the lack of side views of the nanodiscs. Thus, we could not determine decisively the relative orientation of the receptors in this complex.

The dimers described thus far were created using Fzd4–sGFP and LRP6–sGFP constructs that contain 39 and 63 residues, respectively, between the last residue at the membrane and the sGFP fragments, as measured from the last membrane contact in Fzd4 (17) or the last palmitoylated cysteine in LRP6 (18). Assuming full extension of the linkage and 0.35 nm per residue, the interdimer distance in the GFP-tethered Fzd4 homodimer and Fzd4–LRP6 heterodimer would then be 27.3 and 35.7 nm, respectively. Given these distances, antiparallel reconstitution of GFP-tethered dimers into nanodiscs with 13 or 10 nm diameters, as those used here, is theoretically possible for both dimers. We therefore sought to assess the topology of dimers with shortened linkers and produced Fzd4Δ513-sGFP and Fzd4Δ523-sGFP, C-terminally truncated homodimers possessing theoretical interdimer linker lengths of 10.5 and 16.8 nm, respectively. Fab-bound homodimers were assessed by negative stain and 2D classification (Fig. S5). The Fzd4Δ513 homodimer interestingly did not show the same preferred orientation as the full-length Fzd4 homodimer, and 2D classes predominantly displayed two Fabs on the same side of the nanodisc. The Fzd4Δ523 homodimer 2D classes were again dominated by face-on views, but the side views depicted Fabs on the same side of the nanodisc, as with the full-length homodimer.

### Reconstituted receptors bind their extracellular and intracellular partners

**Figure 6:**
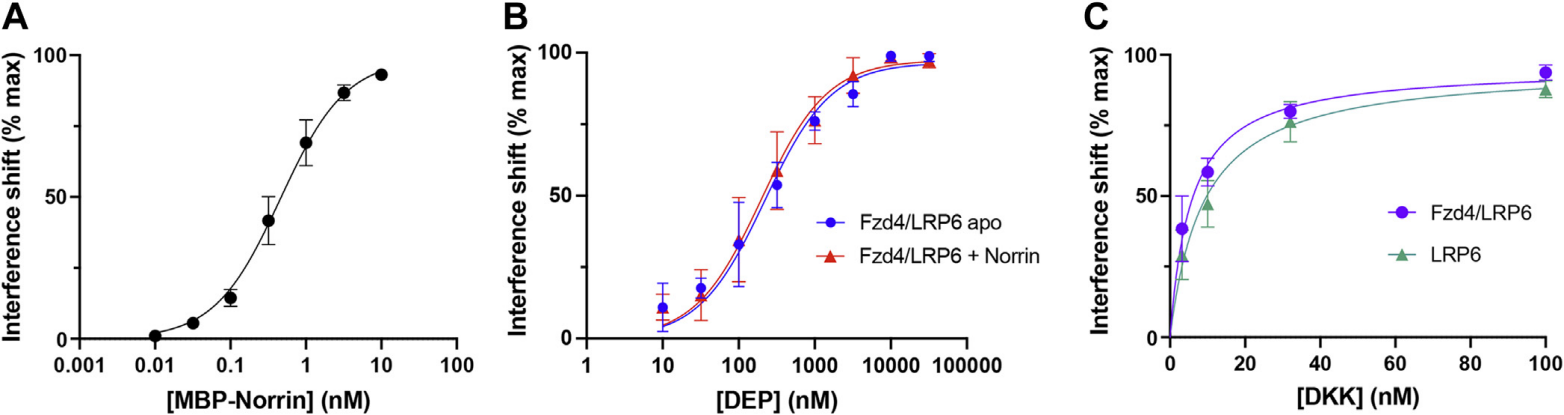
**Reconstituted receptors bind their extracellular and intracellular partners.** Purified reconstituted dimeric receptors bind known ligands by biolayer interferometry. *A*, purified MBP–Norrin binding to Fzd4 homodimer in biotinylated nanodiscs by biolayer interferometry (steady-state *K*_*D*_ = 0.46 nM), n = 3 independent experiments. *B*, purified Dvl2 DEP domain binding to Fzd4–LRP6 heterodimer biotinylated nanodiscs by biolayer interferometry, apo (steady-state *K*_*D*_ = 220 nM) or prebound to Norrin (steady-state *K*_*D*_ = 198 nM), n = 3 independent experiments each. *C*, purified DKK1 binding to Fzd4–LRP6 heterodimer (steady-state *K*_*D*_ = 5.6 nM) or LRP6 (steady-state *K*_*D*_ = 9.0 nM) biotinylated nanodiscs by biolayer interferometry, n = 5 (Fzd4–LRP6) or 4 (LRP6) independent experiments. DEP, contraction of Dishevelled, Egl-10, and Pleckstrin; DKK1, Dikkopf1; Fzd4, Frizzled-4; LRP6, low-density lipoprotein receptor–related protein 6; MBP, maltose-binding protein.

The functionality of the reconstituted receptors was assessed using binding to known partners by biolayer interferometry (Figs. 6 and S6). Fzd4 can bind to Norrin, a secreted protein from the transforming growth factor-beta superfamily that, unlike Wnt proteins, is not modified by lipids, which facilitates quantitative binding analysis. The Fzd4 homodimer bound to Norrin ligand with subnanomolar affinity, similar to monomeric Fzd4 (E. Bruguera, unpublished results). Fzd4–LRP6 heterodimer bound Dishevelled-2 (Dvl2) DEP (contraction of Dishevelled, Egl-10, and Pleckstrin) domain with a steady-state *K*_*D*_ of about 200 nM, which was unaffected by the presence of saturating Norrin ligand, and showed similar affinity and kinetics as Fzd4 alone binding to DEP (J. Mahoney, unpublished results). Fzd4–LRP6 nanodiscs also bound Dikkopf1 (DKK1), an LRP5/6 antagonist, with an affinity of 5.6 nM, similar to previously reported affinities (19, 20, 21).

## Discussion

Here, we present a flexible method to purify homodimers and heterodimers of membrane proteins in nanodiscs with controlled composition, orientation, and stoichiometry. We have validated this method with a homodimer and a heterodimer of receptors with differing sizes and topologies. The fluorescence and stability of the sGFP system enables facile construct screening and provides an additional dimer-specific purification handle.

With appropriate tagging strategies, our purification scheme can be adapted for a variety of membrane proteins. A primary consideration is the relative protein size and the resulting separability of different species by SEC. For dimers with compact soluble domains (*e.g.*, Fzd4 homodimer), dimers reconstituted in one nanodisc can be purified away from GFP-linked protomers embedded in separate nanodiscs but not monomeric receptors using SEC (Fig. 3, *B*–*D*). These reconstituted dimers can then be separated from monomers and empty discs using GFPnb-conjugated resin. For a heterodimeric complex in which the extramembranous region(s) of one protein is significantly larger than the other (*e.g.*, Fzd4–LRP6 heterodimer), the GFP can be proteolytically removed and the heterodimer separated from the smaller component by SEC. Affinity chromatography using a tag on the smaller component allows purification from the larger component (Fig. 4, *B*–*D*). One of these two purification schemes should work for most dimer pairs. Alternatively, cleavage of the sGFP moiety followed by tandem affinity purification to isolate discs containing both desired components can achieve the same result for all systems without SEC.

While the GFP-tethered dimers can be purified in detergent, we observed that tethered Fzd4 receptors occupy separate micelles (Fig. S1*D*). We expect that the tendency of dimers to associate within the same detergent micelle will depend upon the strength of interaction between the protomers and, in some cases, the presence of specific lipids. Reconstitution of dimers in nanodiscs is optimal for probing functional contributions from intramembrane interactions or for structural studies seeking to resolve weak or lipid-mediated intramembrane contacts. Moreover, the nanodiscs allow formation of an approximately native lipid environment of defined composition.

The GFP moiety provides a facile method to estimate yields by SDS-PAGE. The final recovery of dimeric complexes in discs was 5 to 15% (Fig. S3). Compared with the production of nanodisc-embedded monomers, for which we typically see a 50% yield, losses can be attributed to GFP-dimerized species with each protomer in a separate nanodisc, material that is purified away by size exclusion (*e.g.*, for Fzd4 homodimer) and/or affinity chromatography (*e.g.*, for the Fzd4–LRP6 heterodimer). Nonetheless, these yields are sufficient for many biochemical assays as well as structural studies by cryo-EM.

The yield of correctly oriented reconstituted membrane protein dimers may depend on the specific proteins, lipids, disc belt size, and linker lengths used. For example, for the Fzd4 homodimer in this work, we used a 13 nm diameter nanodisc (*i.e.*, MSP1E3D1) that is 4.5 nm thick. In this case, antiparallel reconstitution is theoretically prohibited when the sequences linking the transmembrane domains to the sGFP tags are shorter than 50 residues in total between the two proteins (fully extended to 17.5 nm, assuming 0.35 nm per residue). However, the linker tethering the full-length Fzd4 dimers spanned 78 residues in total, measured from helix eight membrane contacts (17) to the sGFP fragments, and this still produced parallel dimers (Fig. 5), although the existence of a small population of antiparallel dimers cannot be excluded. Homodimers of Fzd4Δ513, with a 10.5 nm linker that theoretically precludes antiparallel reconstitution, indeed showed parallel reconstitution as characterized by negative-stain EM and 2D classification (Fig. S5, *A* and *B*). We expect the upper limit of linker length that yields primarily parallel dimers to be protein dependent. The relative orientation of reconstituted protomers can be evaluated by negative stain, as done here, or by interprotomer FRET of fluorescently labeled dimers (22).

Previous methods have purified parallel nanodisc-embedded dimers using a variety of scaffolds. Parallel reconstituted GPCR homodimers have been obtained using N-terminal peptide tags that bind calmodulin with a 2:1 stoichiometry (22). Calmodulin is used to selectively purify parallel homodimers from antiparallel ones after reconstitution, as opposed to enforcing parallel orientation during reconstitution as done here, resulting in an additional loss. Because the calmodulin-binding peptide tags are identical, this method is also limited to homodimer reconstitution unless tandem affinity purification is employed. Another group has conjugated receptors to complementary DNA strands to enforce parallel reconstitution of dimers and trimers (23). These DNA tethers can yield parallel heterodimers and larger oligomers but require separate purification and labeling steps for each component, as well as minimal-cysteine receptor constructs to allow maleimide-mediated conjugation of the DNA strands. An approach similar to our method was reported using FKBP (the 12-kDa FK506 binding protein) and FKBP12/rapamycin binding–fused receptors dimerized during expression with rapamycin and reconstituted into nanodiscs (24). These structured domains could be a viable alternative to our method if poorly behaved sGFP1–10 hampers expression, although without a dimer-specific purification handle such as the GFPnb resin, this method requires tandem affinity purification. In addition, our sGFP method confers analytical and practical advantages because of its fluorescence and SDS-PAGE stability.

Both dimers purified here were tethered *via* cytosolic C termini. Although we have not tested N-terminal or extracellular tethers, we would expect these to work based on successful complementation of sGFP in other topological contexts (*e.g.*, (25, 26, 27, 28)). Other sGFP constructs may yield additional flexibility in construct design: sGFP10–11 inserted into a loop internal to a fusion protein sequence will complement separately expressed GFP strands 1 to 9 (25, 28). This suggests that sGFP10–11 does not need to be at a terminus of a tethered receptor, providing a way to avoid the truncation of a terminus (*e.g.*, that of LRP6) while minimizing linker length for efficient parallel reconstitution.

The sGFP method may also be extended to form larger defined complexes. For example, tripartite sGFP tags (29) could form ternary complexes using a similar method; an additional GFPnb-tagged component could form a quaternary complex. We have used two different disc sizes (MSP1D1 and MSP1E3D1) in this work, but larger discs could accommodate larger numbers of proteins or those with more transmembrane domains.

While we have shown detergent-solubilized and nanodisc-reconstituted complexes, complexes could also be extracted in styrene maleic acid copolymer to yield styrene maleic acid lipid particles (30, 31), enabling reconstitution of complexes in their native local membrane composition. Parallel complexes could also be inserted into liposomes. Cleavage of the GFP could be used to quantify the relative yields of complexes in each orientation. Under conditions yielding one complex per liposome, GFPnb affinity purification of liposomes could render a preparation of liposome-embedded dimers with controlled orientation.

Nanodisc-embedded dimeric complexes enable biochemical evaluation of dimer function in an approximately native membrane environment. The impact of oligomeric state on basal and ligand-mediated receptor activity can be measured; for example, we have used this method to determine that Fzd4-LRP6 heterodimerization does not impact its ability to recruit the Dvl2 DEP domain, regardless of the presence of ligand (Fig. 6*B*). Cooperativity within homodimeric complexes can be assessed using mutational studies on defined dimers composed of one wildtype and one mutant receptor. Such experiments will allow for a mechanistic understanding of dominant-negative mutations in oligomeric membrane protein receptors and transporters (32). This method also enables structural investigations of weak or transient intermembrane interfaces, as nanodiscs are amenable to single-particle cryo-EM.

## Experimental procedures

### Cloning of receptor constructs

Mouse Fzd4 (residues 42–537), with an N-terminal hemagglutinin signal peptide followed immediately by a FLAG tag, was first subcloned into pcDNA3.1 (Invitrogen). The sGFP sequences were a gift from Steven Boxer (33) and correspond to the sequences “GFP1–10 OPT” and “GFP 11 M3” engineered by the Waldo group (12). Using Gibson Assembly (New England Biosciences), the Fzd4 construct was simultaneously subcloned into pEZT (34) and appended C-terminally with a 1× GS linker, a 3C protease cleavage site, a 2× GS linker, and finally sGFP11. The complementary construct, in which Fzd4 was appended with a 1× GS linker, a 3C protease cleavage site, a 4-residue linker (GGTS), and sGFP1–10, was similarly cloned. Both Fzd4 constructs were further appended with a GS linker sequence followed by residues 534 to 537 of Fzd4 (sequence ETVV), which constitutes a PDZ ligand when at the C terminus. Both constructs were subcloned into pFastBacDual using restriction enzymes NheI and KpnI (Thermo Fisher Scientific) and T4 DNA ligase (New England Biosciences). The C terminus of Fzd4 in both constructs was truncated at residues 513 and 523 using Gibson Assembly.

Human LRP6 (residues 20–1613), with an N-terminal hemagglutinin signal peptide followed immediately by a FLAG tag, and a C-terminal His_8_ tag, was first subcloned into pcDNA3.1 (Invitrogen); this was truncated at residue 1439 by overlap extension PCR. Overlap extension PCR was also used to introduce a BamHI site after the His_8_ tag and before the stop codon. sGFP1–10 and sGFP11 were amplified with primers designed to flank them N-terminally with a BamHI site followed by the same 1× GS linker, a 3C protease cleavage site, and four-residue linkers as in the Fzd4 constructs, and C-terminally with a stop codon followed by a KpnI site. These fragments were inserted into the C terminus of the LRP6 construct using restriction enzymes BamHI and KpnI (Thermo Fisher Scientific) and T4 ligase. Both constructs were subcloned into pEZT using restriction enzymes BspOI and KpnI (Thermo Fisher Scientific) and T4 ligase. The FLAG tag was removed and replaced with a DG linker immediately after the signal peptide, by Gibson Assembly. Full-length untagged human MESD (mesoderm development candidate 2; residues 1–234), an LRP chaperone, was also subcloned into pEZT for coexpression with LRP6 constructs.

Plasmids with an N-terminal signal peptide followed by a FLAG tag and C-terminal cleavable split GFP fragments have been deposited at Addgene (#182931 and 182932).

### Cell maintenance for protein expression

Expi293 cells were used for preliminary construct screening, maintained in Expi293 media at 125 rpm, 37 °C, and 8% CO_2_, and transfected with ExpiFectamine293 (Thermo Fisher Scientific) according to the manufacturer's instructions. Sf9 insect cells were maintained in ESF 921 media (Expression Systems) at 110 rpm and 27 °C. For protein production, Sf9 cells were infected at a density of 3-4 × 10^6^ cells/ml. Freestyle293 cells were maintained in Freestyle293 media (Thermo Fisher Scientific) at 125 rpm, 37 °C, and 8% CO_2_ in baffled flasks and transduced at a density of 1.5 × 10^6^ cells/ml for protein expression by baculovirus for mammalian cells (BacMam).

### Expression testing by Western blot

Sf9 or Expi293 cells were grown in 6-well plates, 2.5 ml culture per well, for small-scale expression tests. Cell pellets from 200 μl of culture harvested 48 or 72 h post-transfection or infection were lysed in hypotonic lysis buffer (50 mM Tris [pH 8.0], 1 mM EDTA, 1× protease inhibitor cocktail), pelleted by centrifugation, and resuspended in solubilization buffer (20 mM Hepes [pH 8.0], 150 mM NaCl, 10% [v/v] glycerol, 1% [w/v] DDM [Anatrace], 0.1% [w/v] cholesteryl hemisuccinate [CHS; Anatrace], and 1× protease inhibitor cocktail) for 1 h with rotation at 4 °C. After centrifugation for 10 min at 15,000*g*, supernatant was mixed with Laemmli sample loading buffer to 1× at room temperature and run on a Stain-Free SDS-PAGE gel (Bio-Rad). The gel was read out on a Gel Doc EZ Imager (Bio-Rad) for Stain-Free fluorescence to verify loading consistency. For sGFP dimer coexpressions, the gels were also imaged for in-gel GFP fluorescence (Gel Doc blue tray), which was sufficient to determine the best expression condition in high-expression cases. For higher signal or for testing expression of individual constructs, gels were transferred to a nitrocellulose membrane, probed with 1 μg/ml M1 mouse anti-FLAG antibody (Sigma), followed by IRDye 800CW goat antimouse antibody (LI-COR), and imaged with a LI-COR Odyssey scanner.

### Expression testing by flow cytometry

A 20 μl sample of cell suspension was transferred to a 96-well v-bottom plate, washed by centrifugation (500*g* for 3 min), and incubated with 0.1 μg/ml M1 anti-FLAG antibody conjugated to Alexa Fluor 647 (Thermo Fisher Scientific) for surface staining in binding buffer (20 mM Hepes, 150 mM NaCl, 1 mg/ml bovine serum albumin [BSA], and 1 mM CaCl_2_). The cells were gently washed two times with binding buffer and finally resuspended in PBS and run immediately on an Accuri C6 flow cytometer. Data were gated for single cells, and M1 anti-FLAG (647 nm) and GFP (488 nm) fluorescence was measured. Median fluorescence was employed to represent expression levels, as mean fluorescence is more sensitive to outlier cells with high nonspecific binding.

### Expression testing by FSEC

10 ml cultures were harvested by centrifugation, resuspended in 1 ml cold hypotonic buffer (50 mM Tris [pH 8.0], 50 mM NaCl, 1 mM EDTA, 1× protease inhibitor cocktail, and DNAse), and lysed by 14 passages through a cell homogenizer (Isobiotec) with 12 μm clearance. The lysate was centrifuged for 5 min at 1000*g* to remove nuclei, then membrane-containing supernatant was centrifuged (15 min × 15,000*g*), supernatant was aspirated, and the membrane pellet was resuspended in 300 μl of solubilization buffer (20 mM Hepes [pH 8.0], 150 mM NaCl, 10% v/v glycerol, 1% w/v DDM, 0.1% w/v CHS, and 1× protease inhibitor cocktail), and rotated for 1 h at 4 °C. After centrifugation for 10 min × 15,000*g*, the supernatant was collected and transferred to a filter vial (catalog no.: 35540; Thomson). A Superose 6 Increase 10/300 column was injected with 100 μl filtered solute using an Ultimate3000 HPLC equipped with an autosampler and a fluorescence detector set to 485 nm excitation/508 nm emission.

### Virus production

Baculovirus was produced and amplified using the Bac-to-Bac system (Thermo Fisher Scientific; pFastBacDual vector) or the BestBac system (Expression Systems; pVL1393 or pAcGP67 vectors) in Sf9 cells according to the manufacturers' instructions. The optimal strength and length of infection for each P2 virus were determined by small-scale Western blots, flow cytometry, and FSEC as described previously.

BacMam (35) was similarly produced from constructs in the pEZT vector and the Bac-to-Bac system. BacMam P2 virus was filtered with a sterile 0.45 μm filter, concentrated by pelleting in autoclaved ultracentrifuge tubes at 120,000*g* for 1 h, and resuspended by pipetting into FreeStyle293 media. Concentrated virus in pEZT was titered as previously described (34), and optimal multiplicity of infection (MOI) ratios were determined by flow cytometry. P2 virus was used within 1 month; subsequent batches were titered and expressed at the predetermined MOI.

### Purification of receptor monomers and dimers

For receptor expression, all lysis, wash, and affinity column buffers included the following protease inhibitors: 0.15 μM aprotinin, 1 μM E-64, 1 μM leupeptin, 200 μM phenylmethylsulfonyl fluoride, 60 μM *N*-p-tosyl-l-phenylalanine chloromethyl ketone, and 60 μM *N*α-tosyl-l-lysine chloromethyl ketone.

Fzd4 monomer and homodimer were expressed in Sf9 cells and harvested 48 h postinfection (1500*g* for 10 min). For Fzd4 monomer purification, baculovirus was generated from the pVL1393 transfer vector containing FLAG-tagged mouse Fzd4 (residues 41–537). Fzd4 homodimer was purified from Sf9 cells coinfected with FLAG-Fzd4-3C-sGFP1–10-PDZ and FLAG-Fzd4-3C-sGFP11-PDZ constructs such that the Fzd4-sGFP11 was expressed in excess.

LRP6 monomer and LRP6–Fzd4 heterodimer were produced in Freestyle293 cells. For the production of monomeric LRP6, baculovirus was generated from human LRP6 (residues 20–1439, with an N-terminal FLAG tag and C-terminal His_8_ tag) and MESD chaperone, both cloned into pEZT vectors, and cells were cotransduced. Fzd4–LRP6 heterodimer constructs LRP6-3C-sGFP1–10, FLAG-Fzd4-3C-sGFP11-PDZ, and MESD were transduced at an MOI of 2, 2, and 1, respectively. For production of both LRP6 monomer and LRP6–Fzd4 heterodimer, expression was carried out at 30 °C. About 24 h post-transduction, 1 M sodium butyrate was added to a final concentration of 10 mM. Cells were harvested 72 h post-transduction (1500*g*, 10 min).

For all receptor preparations, cell membranes were prepared on the day of harvesting. Cells were resuspended in hypotonic lysis buffer (at a maximum of 3 × 10^7^ cells per ml; 20 mM Hepes [pH 8.0], 1 mM EDTA, 10 mM iodoacetamide; no salt for Freestyle cells, or 65 mM NaCl for Sf9 cells), incubated for 30 min at 650 psi, and lysed by nitrogen cavitation (Parr Instrument Company). Nuclei and cell debris were pelleted at 1000*g* for 15 min; supernatant was centrifuged at 200,000*g* in an ultracentrifuge for 40 min at 4 °C. Pelleted membranes were Dounce homogenized 30× to resuspend into high-salt buffer (20 mM Hepes [pH 8.0] and 500 mM NaCl) and centrifuged at 200,000*g* for 40 min at 4 °C. Pelleted membranes were Dounce homogenized 30× into 50 ml low-salt buffer (20 mM Hepes [pH 8.0] and 100 mM NaCl) and frozen dropwise in liquid nitrogen and then stored at −80 °C until use.

The membranes were thawed and adjusted to 5 mg/ml protein (measured by Bradford assay) in a buffer composed of 20 mM Hepes (pH 8.0), 100 mM NaCl, 10% (v/v) glycerol, 1% (w/v) DDM, and 0.1% (w/v) CHS. The membranes were stirred for 1 to 2 h at 4 °C to solubilize and then centrifuged for 1 h at 4 °C, 200,000*g*. The supernatant was adjusted to 3 mM CaCl_2_ and bound to M1 anti-FLAG agarose in batch, rotating for 1 h at 4 °C. The resin was washed with 10 column volumes (CVs) of high salt buffer (20 mM Hepes [pH 8.0], 500 mM NaCl, 0.1% DDM, 0.01% CHS, and 2 mM CaCl_2_) followed by 10 CVs of low salt buffer (20 mM Hepes [pH 8.0], 100 mM NaCl, 0.1% DDM, 0.01% CHS, and 2 mM CaCl_2_). For preparing LRP6 monomer and Fzd4–LRP6 dimer, resin was additionally washed with 10 CVs of low pH buffer (50 mM sodium acetate [pH 5.0], 150 mM NaCl, 0.1% DDM, 0.01% CHS, and 2 mM CaCl_2_) to remove bound MESD chaperone, and 10 CVs of ATP wash (low salt buffer supplemented with 5 mM ATP, 20 mM MgCl_2_, and 50 mM KCl) to remove bound heat shock protein 70. Protein was eluted for 1 h or overnight at 4 °C by rotating in batch with elution buffer (20 mM Hepes [pH 7.5], 150 mM NaCl, 0.05% DDM, 0.005% CHS, 5 mM EGTA, and 100 μM FLAG peptide [GenScript]).

The M1 eluate was concentrated at 3000*g* in a 100 kDa concentrator (Sartorius) to 15 to 30 μM (Fzd4–LRP6 heterodimer) or 50 to 150 μM (Fzd4 homodimer). The final receptor concentration was measured using absorbance at 280 nm on a Nanodrop3000, and GFP concentration was calculated using an extinction coefficient at absorbance at 485 nm of 37,700 M^−1^ cm^−1^ (36). Aliquots were frozen in liquid nitrogen until used in reconstitutions.

For all monomer purifications, as well as for purified Fzd4 homodimer in detergent, concentrated M1 anti-FLAG eluate was further purified by injection onto a Superose 6 Increase 10/300 column. 250 μl fractions were collected and analyzed by SDS-PAGE, and peak fractions containing monomer or dimer were concentrated and frozen in liquid nitrogen.

### SDS-PAGE analysis of sGFP dimer SDS resistance

SEC-purified Fzd4 homodimers in buffer containing glycodiosgenin (Anatrace) concentrated to 37 μM were verified to be pure dimer by agreement between protein concentration calculated using absorbance at 280 nm (using an extinction coefficient calculated by protein sequence on the ExPASy ProtParam tool) and absorbance at 485 nm (using an extinction coefficient 37,700 M^−1^ cm^−1^). The homodimers were diluted to 1.85 μM dimer in pH 8 buffer (20 mM Hepes [pH 8.0], 100 mM NaCl, 1 mM EDTA, and 0.1% glycodiosgenin). 6 μl of diluted dimer was mixed with 6 μl 2× Laemmli loading buffer (100 mM Tris–HCl [pH 8.0], 4% w/v SDS, 20% v/v glycerol, 0.1% w/v bromophenol blue, and 5% v/v β-mercaptoethanol) for the indicated amount of time, starting with the longest time points, such that all time points ended simultaneously. Incubations were performed at room temperature, in a 37 °C incubator, or in a heat block set to 50 °C. For the low pH condition, 6 μl of diluted dimer was mixed with 1.2 μl of 1 M glycine (pH 2.7) and then after 2 min, mixed with 3 μl 4× loading buffer and 2.4 μl 1 M Tris (pH 8.5) immediately before gel loading.

### Reconstitution and purification of receptor-containing nanodiscs

Reconstitution was performed according to previously published protocols (37, 38) using 16:0 to 18:1 phosphatidylcholine (POPC) and phosphatidylglycerol as well as 18:0 to 20:4 brain phosphatidylinositol-4,5-bisphosphate (PIP_2_) purchased from Avanti Polar Lipids, and cholesterol (Sigma), all predissolved in organic solvent. A 48:32:20 molar mixture of POPC:phosphatidylglycerol:cholesterol was predominantly used; alternatively, a 75:5:20 mixture of POPC:PIP_2_:cholesterol was used in nanodiscs used for DEP domain binding. Lipids were transferred to glass tubes, dried under a stream of argon and left under vacuum for 1 h, and then solubilized with HNE buffer (20 mM Hepes pH 8.0, 100 mM NaCl, 1 mM EDTA) supplemented with 50 mM sodium cholate. HNE buffer, receptor, and MSP were added to reach final concentrations of 18 mM sodium cholate, 6 mM lipid, 0.1 mM MSP1D1 or 0.07 mM MSPE3D1, and 5 μM receptor for monomeric reconstitutions. For dimeric reconstitutions, final concentrations were 18 mM sodium cholate, 6 mM lipid, 0.07 mM MSPE3D1 or 0.1 mM MSP1D1, and 7 μM receptor dimer. Reconstitution mixture, typically 100 μl, was chilled on ice for 1 h, then transferred to an Eppendorf containing methanol-activated and equilibrated Bio-Beads (Bio-Rad; 83 mg beads per nmol of lipids), and incubated overnight on a nutator at 4 °C for detergent removal. Solution containing nanodiscs was recovered by pipetting from the Bio-Beads, and one additional volume of buffer used to wash the Bio-Beads was collected.

For monomeric reconstitutions, nanodiscs were injected onto a Superose 6 Increase 10/300 column equilibrated in HNE buffer. Peak receptor–containing fractions, as determined by SDS-PAGE, were supplemented with CaCl_2_ to 1.5 mM and bound to 100 μl M1 anti-FLAG affinity resin, which was washed with 10 CVs of FLAG buffer (20 mM Hepes [pH 8.0], 100 mM NaCl, and 1 mM CaCl_2_). Receptor-containing nanodiscs were eluted with HNE supplemented with 5 mM EGTA and 100 μM FLAG peptide. For receptors to be used in binding assays, wash and elution buffer was supplemented with 1 mg/ml BSA to prevent sticking and increase yield. BSA was omitted for nanodiscs analyzed by negative-stain EM.

Reconstituted Fzd4 homodimer in MSPE3D1 nanodiscs was injected onto a Superose 6 Increase 10/300 column equilibrated in HNE buffer. Fractions were analyzed by SDS-PAGE and fluorescence: 10 μl of each fraction was transferred to a black 384-well plate for GFP fluorescence analysis on a Synergy2 plate reader equipped with 485/20 excitation and 528/20 emission filters (BioTek). Peak fractions containing reconstituted GFP-tethered dimers were pooled, with care taken to avoid left shoulder fractions containing GFP-tethered species with protomers reconstituted into separate nanodiscs, and bound to 100 μl GFPnb affinity resin. Resin was washed with HNE buffer and then eluted by on-column cleavage overnight in two CVs of HNE supplemented with 5 μg of 3C protease per 100 μg membrane protein. Wash and elution buffers were supplemented with 1 mg/ml BSA when discs were to be used in binding assays downstream.

For Fzd4–LRP6 heterodimer reconstitutions in MSP1D1 nanodiscs, after detergent removal, the sGFP tag was cleaved by addition of 1 μg of 3C protease per 100 μg membrane protein for 1 h at room temperature. Nanodiscs were purified by Superose 6 Increase and M1 anti-FLAG affinity resin columns as for the monomeric reconstitutions.

The eluted nanodiscs were run on SDS-PAGE along with a standard curve of known amounts of MSP, and concentration was thus quantified using densitometry in ImageJ (imagej.nih.gov).

### Yield quantification by SDS-PAGE

Nanodiscs at various stages of the purification process were analyzed by Stain-Free SDS-PAGE, and quantifications were conducted in ImageJ. The loaded volume for each step represented 1% of the total volume at that given step, except for the final elution fraction, where 5% was loaded for enhanced signal. The molar percent of initial dimer at each step was calculated based on densitometry, normalized to the number of tryptophan residues in each species, as protein fluorescence in Stain-Free gels is tryptophan dependent. Four steps were quantified: (1) the initial reconstitution mixture pre-biobead incubation, (2) the reconstituted nanodiscs after removal from Bio-Beads, (3) the pooled SEC fractions, and (4) the final discs eluted from affinity resin. For the Fzd4 homodimer, the initial amount of protein, amount recovered from Bio-Beads, and amount recovered after SEC were quantified using the intensity of the upper Fzd4 sGFP dimer band, and the final yield was quantified using the intensity of the cleaved Fzd4 band. For the Fzd4–LRP6 heterodimer, the initial amount of protein and the amount recovered from beads was quantified using the intensity of the upper heterodimer sGFP band. The amount recovered from SEC was quantified using the Fzd4 band because free LRP6 comigrates with the heterodimer on SEC. The final yield was quantified using the LRP6 band, as Fzd4 comigrates with BSA on SDS-PAGE.

### Assessment of stoichiometry by Western blot

Purified receptors and MSP protein, with concentrations determined by absorbance at 280 nm using a Nanodrop3000, were used to create standard curves by serial dilution. Standards were loaded alongside nanodisc samples on SDS-PAGE and transferred to a nitrocellulose membrane. M1 anti-FLAG antibody (Sigma) was used to detect FLAG-tagged Fzd4, and THE His Tag antibody (GenScript) was used to detect His_6_-tagged MSP1D1 and MSPE3D1 as well as His_8_-tagged LRP6. For Fzd4 quantification, the standard curve consisted of Fzd4 in detergent at 0.5, 1, 2, 4, and 8 nM, with 10 μl loaded per lane. All His-tag standards were combined on the same gel, with lanes containing 10 μl each of 12.5, 25, 50, 100, and 200 nM LRP6, as well as 25, 50, 100, 200, and 400 nM each MSP. Nanodisc samples were typically diluted fourfold for the anti-His blot and 100-fold for the anti-FLAG blot to fall within the linear range of the standard curves. After incubation with primary antibody by rocking at 4 °C overnight followed by incubation with 1:15,000 IRDye 800CW goat antimouse antibody (LI-COR), the blots were imaged with an Odyssey LI-COR scanner, and band intensities were quantified using the LI-COR software. Standard band intensities were plotted against concentration, and standards beyond the linear range were excluded from analysis; for all blots, quantified sample bands were verified to fall within the linear range given by the standard curve. After normalizing all band intensities to the highest concentration of the respective standard curve on the corresponding blot, average standard curves were created in Excel and used to calculate the concentration of each component within each sample. At least three independent reconstitutions representing at least two different dimer preparations were each measured in duplicate; the standard deviation between samples is reported.

### Negative stain and processing for orientation analysis

A Fab directed against the extracellular domain of Fzd4 (16) was bound to nanodisc samples during the final affinity step purification. Receptor-bound, washed, GFPnb (for the Fzd4 homodimer), or M1 anti-FLAG (for the Fzd4–LRP6 heterodimer) resin was supplemented with two CVs of wash buffer containing 5 μM Fab and incubated rotating for 30 min at room temperature. Resin was washed with another 10 CVs of buffer and eluted as aforementioned. CF-300Cu grids (Electron Microscopy Sciences) were glow discharged (PELCO easiGlow) at 15 mA for 40 s. Grids were overlaid with a 3.5 μl drop of buffer containing nanodiscs diluted to ∼0.004 mg/ml for 30 to 60 s, then blotted with filter paper (Whatman), and washed three times with 5 μl 1% uranyl acetate. Grids were imaged on a 100 kV Morgagni electron microscope equipped with an Orius CCD camera (Gatan) at 40,000×. For all samples, 50 images were taken and imported into cryoSPARC (Structural Biotechnology Inc., version 3.2.0). Particles from five micrographs were manually picked and subjected to 2D classification. Centered featureless 2D classes were used as templates for autopicking from the 50 images. The autopicked particles were subject to a first round of 2D classification; particles from 2D classes with single centered particles were selected and subject to a second round of 2D classification. Particles were reextracted using the aligned shifts from the second round of 2D classification to recenter particles.

For Fzd4 homodimer classification, side views were examined in more detail as follows. Re-extracted particles from the second round of classification were subject to a third round of 2D classification, in which particles were windowed with a small radius (15–18 nm) such that the alignment and classification was dominated by the nanodisc. Classes with ovular nanodiscs indicative of side views were selected and subjected to a fourth round of 2D classification with a larger radius such that the Fabs were a visible factor in the alignment.

For Fzd4–LRP6 heterodimer classification, re-extracted particles from the second round of classification were subjected to a third round of 2D classification. Again, 2D classes with single centered particles were selected and subjected to a fourth round of 2D classification.

### β-catenin transcriptional reporter assay

Wildtype human embryonic kidney 293T cells maintained in Dulbecco's modified Eagle's medium supplemented with 10% (v/v) fetal bovine serum (Gemini) were seeded on white 96-well plates (PerkinElmer) at 7500 cells/well. About 24 h later, cells were transfected with receptor vectors (1 ng each/well), Super8xTOPFlash (Addgene plasmid no.: 12456; 80 ng/well), and LacZ under a cytomegalovirus promoter (20 ng/well) using Lipofectamine 2000 (Invitrogen) according to the manufacturer's instructions. About 16 to 20 h post-transfection, media were replaced with L-cell control or Wnt3a-conditioned media. Cells were lysed 22 to 26 h later, and the Dual-Light system (Invitrogen) was used according to the manufacturer's instructions to quantify luciferase and β-galactosidase activity using a plate reader (BioTek Synergy2).

### Soluble protein purifications

His_6_-tagged MSP1D1 and MSPE3D1 (Addgene plasmid nos.: 20061 and 20066) were expressed in BL21(DE3)-RIL *E. coli* cells and purified as previously described (38, 39). After dialysis of purified MSP protein into buffer containing 20 mM HEPES, pH 8.0, 100 mM NaCl, and 1mM EDTA, protein concentration was determined by A_280_ and an equimolar amount of NHS-PEG4-biotin (ThermoFisher) was added. Following a 30-min incubation at room temperature, the biotinylation reaction was quenched by the addition of 1 M Tris-HCl, pH 8.0, to reach 20 mM final Tris concentration. The mixture was subjected to SEC on Superdex 200 HiLoad 16/600 (Cytiva) in a buffer composed of 20 mM HEPES-NaOH, pH 8.0, 100 mM NaCl, 1 mM EDTA, and 5 mM sodium cholate. Fractions containing MSP were pooled and concentrated to approximately 14 mg/ml (MSP1D1) or 12 mg/ml (MSP1E3D1). Extent of MSP biotinylation was assessed using the Pierce Biotin Quantitation Kit (ThermoFisher). Aliquots of protein were flash-frozen in liquid nitrogen and stored at −80 °C until use.

The His_6_-tagged GFPnb sequence (40) was cloned into pET26(+) and expressed in the periplasm of BL21(DE3)-RIL *E. coli*. and purified according to previously published protocols (41). The nanobody was coupled to cyanogen bromide–activated Sepharose (Cytiva) according to the manufacturer's instructions.

His_6_-tagged full-length human DKK1 in the pAcGP67 transfer vector was purified from baculoviros-infected Sf9 cells according to previously published work (42).

Norrin (residues 33-133), with an N-terminal maltose-binding protein (MBP) tag followed by a 3C protease cleavage site, as well as a C-terminal 1D4 tag, was also subcloned into the pAcGP67 vector. Norrin was purified from Sf9 supernatant harvested 72 h after infection. Filtered media was loaded onto Amylose resin, which was then washed with five bed volumes of wash buffer (20 mM HEPES-NaOH pH 8.0, 150 mM NaCl, 1 mM EDTA, 5% glycerol), which was then supplemented with 10 mM maltose for elution. Eluted MBP-Norrin was concentrated using a 30-kDa Amicon spin concentrator, loaded onto a Superdex 200 Increase 10/300 GL, and eluted in wash buffer. Peak fractions were pooled and used for binding or signaling assays without further concentration. To remove the MBP tag prior to use in assays, MBP-3C-Norrin was diluted to 1 µM and incubated with 3C protease for 30 min at room temperature.

The DEP domain from mouse Dishevelled 2 (residues 416-510) was subcloned into a modified pCDFduet vector with N-terminal His_6_ and MBP tags followed by a TEV protease cleavage site and the DEP sequence. This construct was expressed in *Escherichia coli* strain BL21(DE3)-RIL (Agilent) grown in terrific broth and induced at *A*_600_ of 0.8 with 0.5 mM IPTG and then incubated shaking overnight at 18 °C. Cells were collected by centrifugation, washed once in PBS, and stored at -80 °C until purification. Thawed cell pellets were resuspended in lysis buffer (50 mM Tris-HCl pH 8.0, 150 mM NaCl, 1 mM EDTA, 0.01% Tween-20, 5 mM DTT, protease inhibitors) for lysis by two passes through an Emulsiflex system (Avestin) pulsing at 15,000 psi. The lysate was clarified by centrifugation at 50,000 *g* for 30 min and loaded onto Amylose resin (New England Biolabs). The column was washed with ten bed volumes of wash buffer (20 mM HEPES-NaOH pH 8.0, 150 mM NaCl, 1 mM EDTA, 2 mM DTT). Protein was eluted overnight using 0.5 mg of TEV protease in one bed volume of wash buffer at 4 °C with gentle rotation. The eluate was concentrated using a 3-kDa cutoff Amicon spin concentrator and loaded onto a HiLoad Superdex 75 26/600 (Cytiva) in a buffer composed of 20 mM HEPES-NaOH, pH 8.0, 150 mM NaCl, and 0.1 mM TCEP. Peak fractions were pooled, concentrated to ~5 mg/ml protein using a 3-kDa cutoff spin concentrator, and supplemented with glycerol to 10% v/v final concentration. Aliquots of protein were frozen in liquid nitrogen and stored at −80 °C

### Biolayer interferometry

Octet RED384 (Sartorius) or GatorPrime (Gator Bio) instruments were used for biolayer interferometry binding assays. All binding assays were performed at 25 °C with 1000 rpm shaking, in buffer composed of 20 mM Hepes (pH 7.4), 150 mM NaCl, 1 mM EDTA, and 1 mg/ml BSA. For MBP–Norrin and DKK1 binding, biotinylated nanodiscs were diluted to 20 nM for loading onto prehydrated streptavidin biosensors for 3 min, which typically resulted in an interference shift of 2 to 2.5 nm. After loading, tips were dipped into binding buffer for 5 min or until the baseline was flat for all tips.

For MBP–Norrin binding, eight tips were dipped in parallel into wells containing 0, 0.01, 0.032, 0.1, 0.32, 1, 3.2, or 10 nM MBP–Norrin diluted in binding buffer to associate for 3 h, followed by dissociation in binding buffer for an additional 2 h. The 0 nM condition was subtracted from all traces to correct for drift. No detectable binding of 10 nM MBP–Norrin to empty nanodiscs was seen, so no empty nanodisc condition was subtracted.

For DKK1 binding, tips were dipped in parallel into 0, 1, 3.2, 10, 32, or 100 nM DKK1 diluted in binding buffer for an association step of 10 min, then dipped in binding buffer for a dissociation step of 10 min. In parallel, empty discs were dipped in the same conditions, and signal was subtracted; DKK1 showed significant background binding to empty nanodiscs above 32 nM, which hampered efforts to fill out a steady state binding curve at high concentrations, as the background subtraction is imperfect.

For Dvl2 DEP binding to Fzd4–LRP6 heterodimers, high-sensitivity SMAP biosensors (Gator Bio) were loaded with 20 nM nanodiscs containing 5% PIP_2_ for 5 min for an interference shift of 10 nm, which was optimized to ensure sufficient DEP-binding signal while avoiding kinetic artifacts due to crowding of nanodiscs on the biosensor surface. Repeated association–dissociation cycles were performed using the same tip using progressively higher concentrations of DEP in each association step ranging from 0.01 to 32 μM DEP. Association and dissociartion steps were 5 min each, ensuring complete DEP dissociation before the next association–dissociation cycle commenced. The first association–dissociation cycle, used to define linear baseline drift, was performed in wells containing binding buffer without ligand. A reference sensor loaded with a matched sample of empty nanodiscs was run in parallel to define and subtract nonspecific binding.

Preliminary processing, including step alignment, Savitzky–Golay filtering, and signal subtraction of control conditions, was performed in Octet Data Analysis 10.0 (Sartorius) or Gator 1.7 (Gator Bio) software. Curve fitting was performed in Prism (GraphPad Software, Inc) using single-phase association and dissociation models. The observed association rate constant (*K*_obs_) was plotted as a function of ligand concentration and fit to a straight line, the slope of which was taken as the *K*_on_. The highest concentrations of DKK1 (100 nM) and DEP (10 and 32 μM) were excluded from the *K*_on_ calculation because the *K*_obs_ was no longer linear in that regime, perhaps because of crowding effects that became evident with fast kinetics. *K*_off_ was determined by averaging the dissociation rate constants given by dissociation curve fits at multiple concentrations. Steady-state binding affinity values were obtained by fitting equilibrium data (signal plateau) to a one-site–specific binding model.

Each binding experiment was performed in triplicate, with replicates representing at least two independent preparations of receptor and at least two independent preparations of binding partner. For averaging of equilibrium data, binding data were normalized to the maximum signal plateau value for each replicate.

## Data availability

All data are contained within the article.

## Supporting information

This article contains supporting information.

## Conflict of interest

The authors declare that they have no conflicts of interest with the contents of this article.
