# Supplementary material for: Reconstitution of purified membrane protein dimers in lipid nanodiscs with defined stoichiometry and orientation using a split GFP tether
Source: J Biol Chem. 2022 Jan 22;298(4):101628. doi: 10.1016/j.jbc.2022.101628 (PMC8980801; doi:10.1016/j.jbc.2022.101628)
Supplement: Supplemental Figures S1–S6 [file mmc1.pdf]

Supporting Information

Reconstitution of purified membrane protein dimers in lipid nanodiscs with defined stoichiometry and orientation using a split GFP tether

Elise S. Bruguera, Jacob P. Mahoney, and William I. Weis†

Departments of Molecular & Cellular Physiology and Structural Biology,
Stanford University School of Medicine; Stanford, CA 94305

†*Address correspondence to weis@stanford.edu*

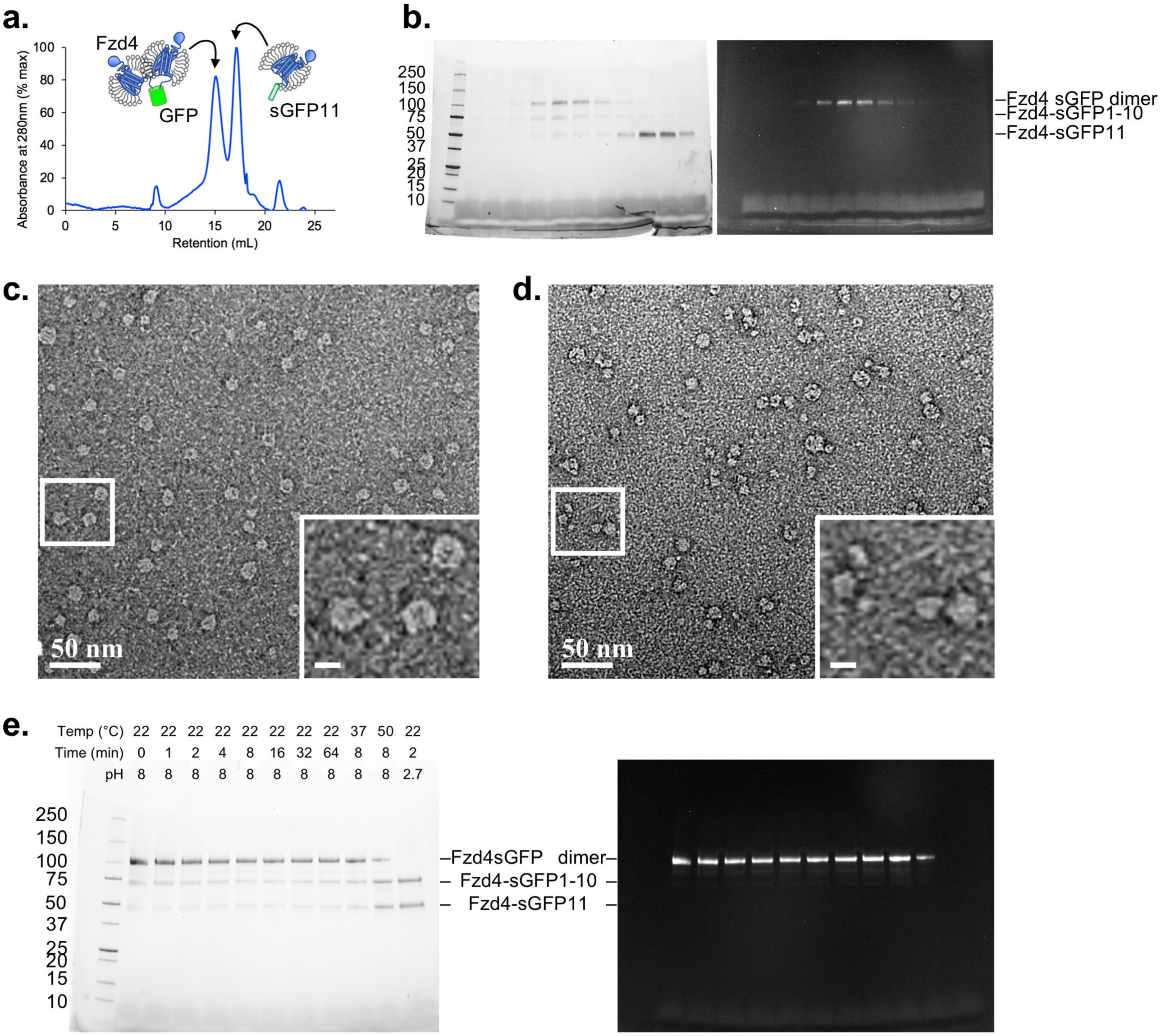

Figure S1: Purification of Fzd4 homodimer in detergent. **(a)** FLAG-Fzd4-sGFP1-10 was co-expressed with excess FLAG-Fzd4-sGFP11 purified in DDM on M1 anti-FLAG affinity resin, then injected onto a Superose 6 10/300 column in 0.02% DDM buffer, where two peaks are resolved. Cartoons indicate dimeric vs monomeric species comprising each peak according to **(b)** SDS-PAGE gel. Equally spaced fractions eluting between 13 and 18 mL were analyzed on a Stain-Free gel at *left*, which shows that the left peak is predominantly intact GFP complex (the dimer is resistant to SDS), and that the right peak is excess free Fzd4-sGFP11 monomer. At *right*, in-gel GFP fluorescence imaging of the same gel verifies that the top band contains intact GFP. **(c)** Negative stain EM of center fraction of Fzd4 monomer-containing second peak (17.5 mL retention in (a), lane 9 in (b)) shows single micelles, in contrast to **(d)** micrograph of center fraction of GFP dimer-containing first peak (15 mL retention in (a), lane 5 in (b)) shows micelle “doublets” stuck together, suggesting that the GFP-dimerized Fzd4 protomers occupy separate micelles when in DDM. Scale bars in full micrographs are 50 nm and inset scale bars are 10 nm. **(e)** Intact, SEC-purified Fzd4-GFP dimer in detergent from left peak in (a) was incubated with 1x SDS-PAGE sample buffer for the indicated time at the indicated temperature and pH, then run on a Stain-Free SDS-PAGE gel (*left*) to assess split-GFP stability; the same gel was also imaged using in-gel GFP fluorescence (*right*).

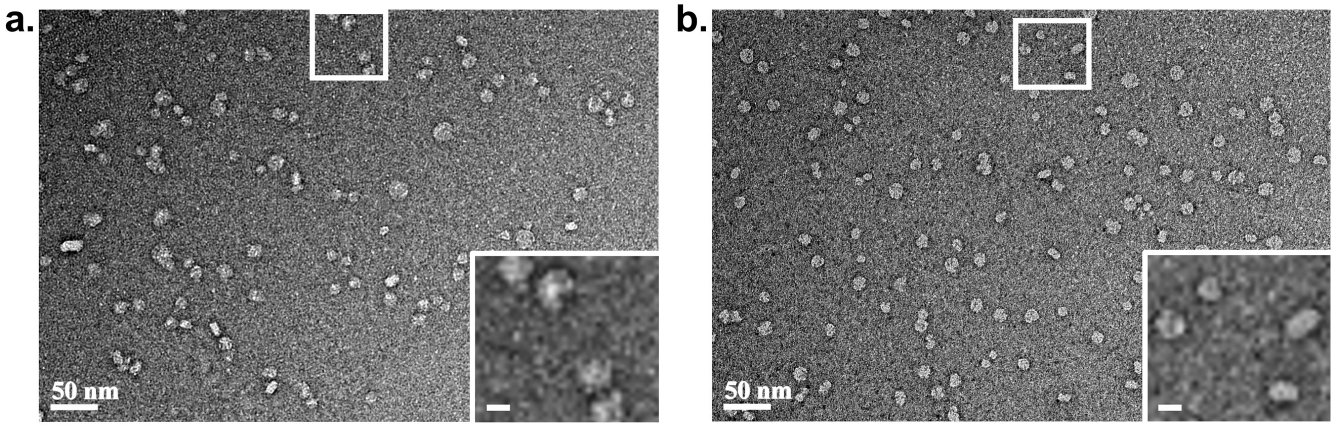

Figure S2. Nanodisc-embedded Fzd4 homodimers separate from GFP-dimerized Fzd4 with each protomer in a separate nanodisc by SEC. (a) Negative-stain EM of peak fraction from left GFP peak shows predominantly “doublet” nanodiscs, or separately-reconstituted but GFP-tethered protomers. **(b)** Negative stain EM of peak fraction from right GFP peak shows predominantly single nanodiscs. Micrograph scale bars are 50 nm, and inset scale bars are 10 nm.

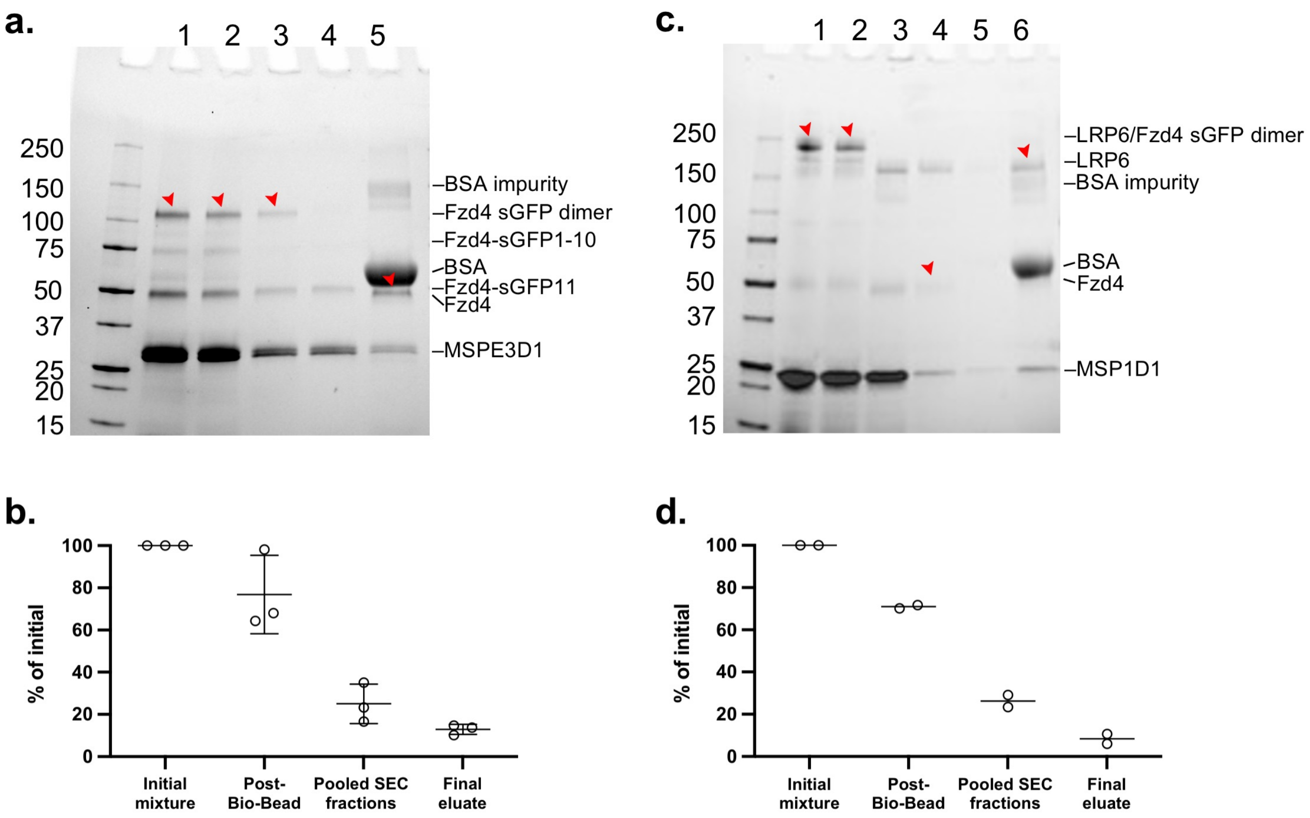

Figure S3: Yield quantification by densitometry. Nanodiscs at various stages of the purification process were run on SDS-PAGE. Each lane contains 1% of the total preparation at the given step, except for the final elution fraction, where 5% was loaded for increased signal. **(a)** Representative gel for the quantification of Fzd4 homodimer yield. Lane: (1) reconstitution mixture before Bio-Bead incubation, (2) reconstitution mixture after removal from Bio-Beads, (3) pooled SEC fractions, (4) GFPnb resin flowthrough, and (5) final eluate off of GFPnb resin, with BSA. Red arrows indicate the bands used for quantification of yield at a given step. **(b)** Quantified average yield ± s.d., as a molar % of initial GFP-receptor dimers obtained by densitometry of gel in (a), at four steps within the purification process: the initial reconstitution mixture pre-Bio-Bead incubation, the reconstituted nanodiscs after removal from Bio-Beads, the pooled SEC fractions, and the final discs eluted from affinity resin. Data represent three independent reconstitutions. The average overall yield was 12.9%. **(c)** Representative gel for quantification of Fzd4/LRP6 heterodimer yield. Lanes: (1) reconstitution mixture pre-biobead incubation (2) reconstitution mixture aver removal from Bio-Beads, (3) cleaved reconstitution mixture, (4) pooled SEC fractions, (5) M1 anti-FLAG resin flowthrough, and (6) final eluate off M1 anti-FLAG resin, with BSA. Red arrows indicate the bands used for quantification of yield at a given step. **(d)** Quantification, as in (b), for two independent Fzd4/LRP6 heterodimer reconstitutions. Yields at each step were generally similar between the homo- and heterodimer preparations, although the overall yield of 8.3% was lower for the heterodimer.

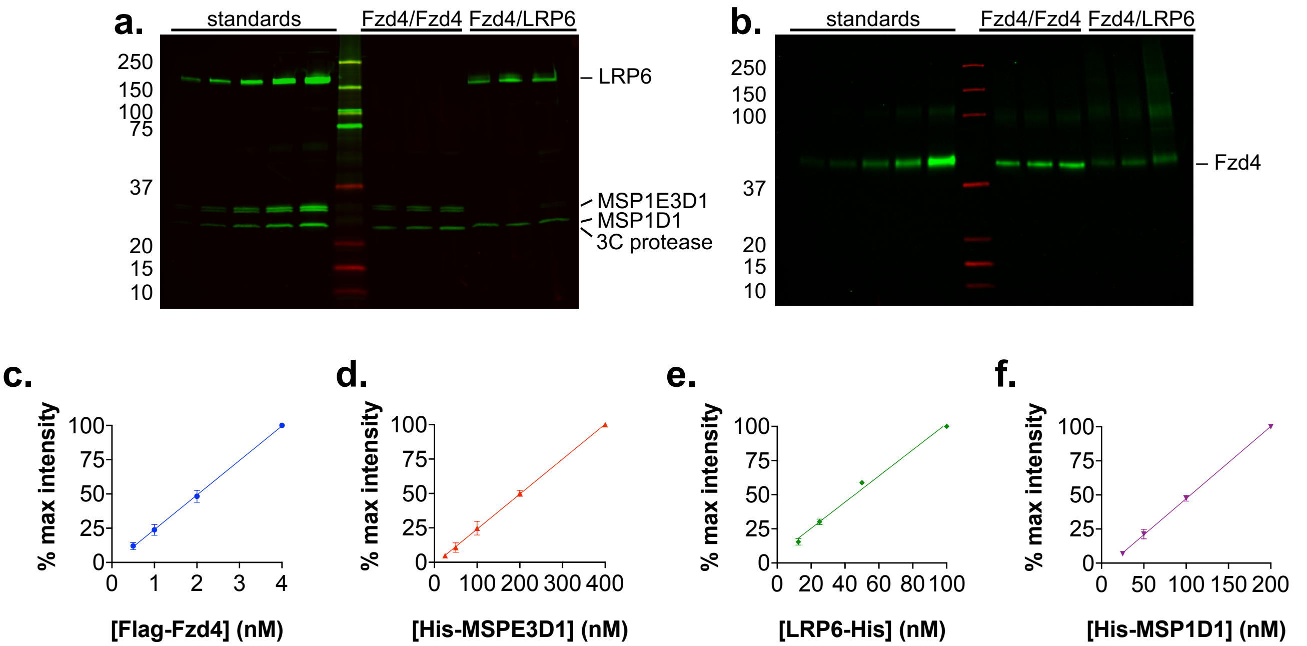

Figure S4: Dimers are stoichiometrically incorporated into nanodiscs. (a) Representative anti-His_6 blot for quantification of His_6-tagged MSPs and LRP6-His_8. Lanes 1-5 contain LRP6-His_8, His_6-MSPE3D1 and His_6-MSP1D1 standards (loaded at 12.5, 25, 50, 100, 200 nM LRP6, and 25, 50, 100, 200, 400 nM each MSP, respectively). Lane 6 contains the molecular weight standards, lanes 7-9 contain Fzd4 dimer nanodiscs, and lanes 10-12 contain Fzd4/LRP6 nanodiscs. 10 µL standard or sample was loaded per lane. **(b)** Representative M1 anti-FLAG blot for quantification of Fzd4, lanes 1-5 contain FLAG-tagged Fzd4 standard (loaded at 0.5, 1, 2, 4 and 8 nM, respectively). Lane 6 contains the ladder, lanes 7-9 contain Fzd4 dimer (MSPE3D1) nanodiscs (and 3C protease, from final affinity purification), and lanes 10-12 contain Fzd4/LRP6 (MSP1D1) nanodiscs. Sample nanodiscs were diluted 1:25 from the anti-His blot loading concentration in (a) to fall within the linear range for M1 anti-FLAG blotting. **(c-f)** Standard curves for calculating concentration of Fzd4, MSPE3D1, LRP6, and MSP1D1, respectively, normalized to intensity of highest concentration within the linear range. In all blots, sample bands fell within the linear range.

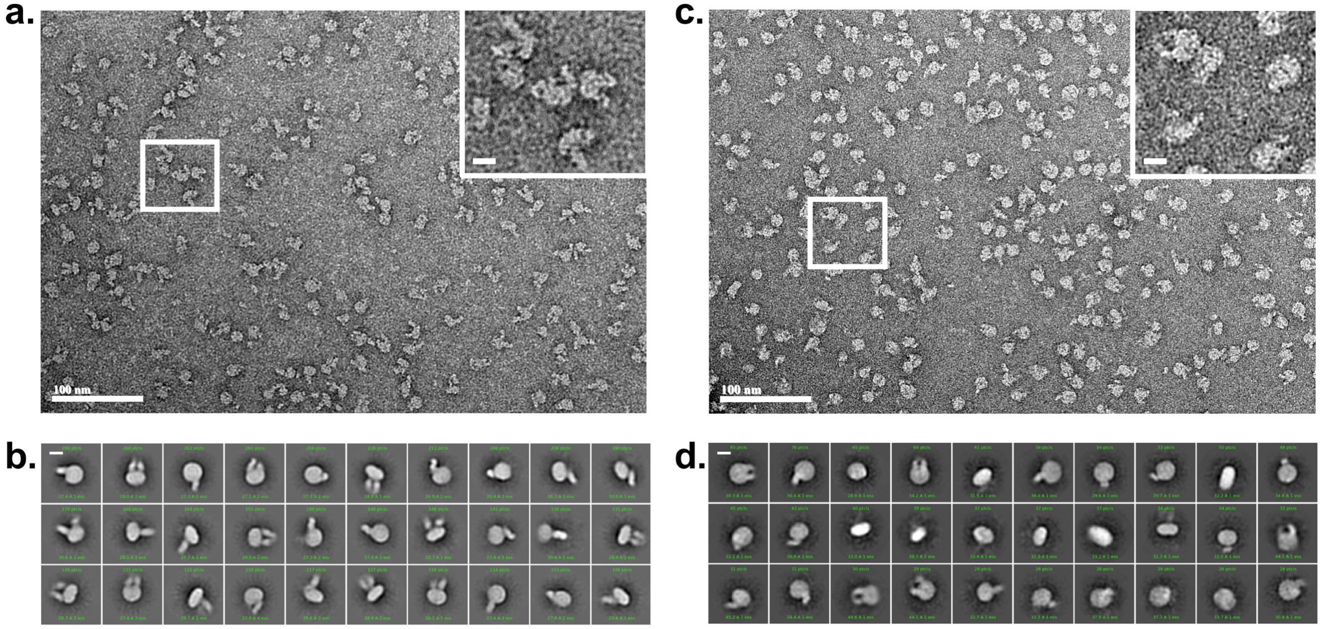

Figure S5: C-terminal truncation of Fzd4 also yields homodimers that are in a parallel orientation in nanodiscs, as evaluated by negative stain electron microscopy. (a) Representative negative stain micrograph and **(b)** 2D class averages of dimeric Fab-bound Fzd4Δ513 (truncated just after helix 8) in MSP1E3D1. Interestingly, more side views were obtained than for full-length Fzd4 dimers (fig. 5b), suggesting that the Fzd4 C terminus contributed to the nanodisc preferential orientation. **(c)** Representative negative stain micrograph and **(d)** 2D class averages of Fab-bound Fzd4Δ523 in MSP1E3D1. These displayed similar orientation preference to full-length Fzd4 dimers. Both Fzd4Δ513 and Fzd4Δ523 should theoretically prohibit antiparallel reconstitution due to the short length of the receptor-sGFP linkers. Scale bars on micrographs are 100 nm, and scale bars in insets and 2D class averages are 10 nm.

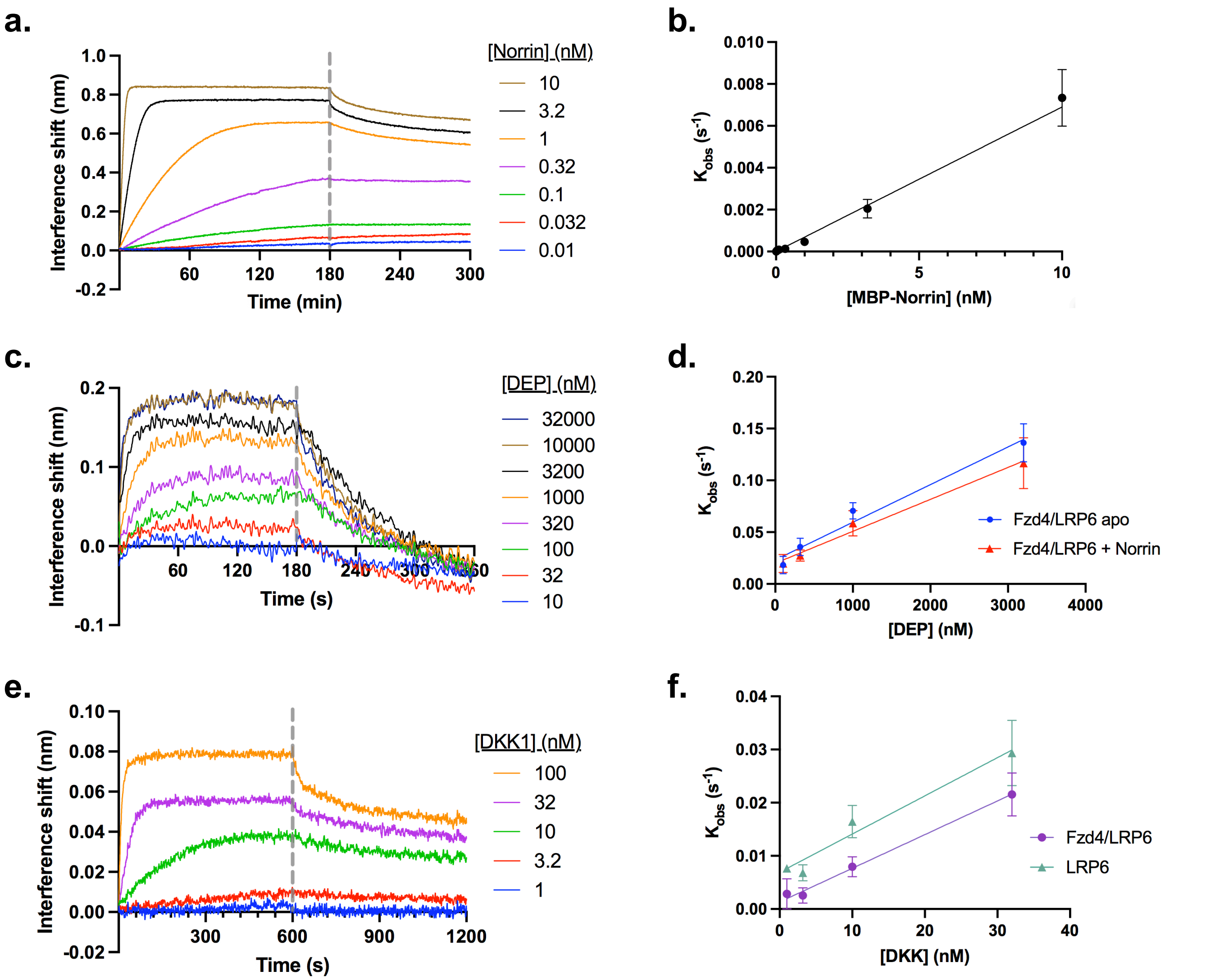

Figure S6: Reconstituted receptors bind their extra- and intracellular partners. Biolayer interferometry traces and rate constants corresponding to binding data in fig. 6. **(a)** BLI trace of maltose-binding protein (MBP)-fused Norrin binding to Fzd4 homodimer. Dissociation was slow, and therefore a significant amount of ligand remained bound at the time when the dissociation step was terminated, so the curves do not return to baseline. **(b)** k_{obs} vs [MBP-Norrin] plot for MBP-Norrin association to Fzd4 homodimer (R^2 = 0.90, k_{on} = 0.00069 nM^{-1} s^{-1}, k_{off} = 0.00034 s^{-1}, kinetic K_D = 0.49 nM, n=3) in fig. 6a. **(c)** Representative BLI trace of DEP domain binding to Fzd4/LRP6 heterodimer. **(d)** k_{obs} vs [DEP] plot for DEP association to Fzd4/LRP6 heterodimer without Norrin (R^2 = 0.97, k_{on} = 0.000036 nM^{-1} s^{-1}, k_{off} = 0.0095 s^{-1}, kinetic K_D = 260 nM) or in the presence of Norrin (R^2 = 0.99, k_{on} = 0.000031 nM^{-1} s^{-1}, k_{off} = 0.013 s^{-1}, kinetic K_D = 420 nM) in 6b. n=3 replicates each. (Data reproduced from (13)). **(e)** BLI trace of DKK1 binding to Fzd4/LRP6 heterodimer. As with Norrin binding in (a), dissociation was slow and the dissociation step was terminated at a time when ligand remained bound, so the curves do not return to baseline. **(f)** Fitted k_{obs} vs [DKK] for DKK association to LRP6 monomer (R^2 = 0.61, k_{on} = 0.00072 nM^{-1} s^{-1}, k_{off} = 0.012 s^{-1}, kinetic K_D = 16.7 nM; n=4) or Fzd4/LRP6 heterodimer (R^2 = 0.69, k_{on} = 0.00063 nM^{-1} s^{-1}, k_{off} = 0.0057 s^{-1}, kinetic K_D = 9.0 nM; n=5) in fig. 6c.
